# Neuronal and Glial Distribution of Tau Protein in the Adult Rat and Monkey

**DOI:** 10.3389/fnmol.2021.607303

**Published:** 2021-04-27

**Authors:** Nicholas M. Kanaan, Tessa Grabinski

**Affiliations:** ^1^Department of Translational Neuroscience, College of Human Medicine, Michigan State University, Grand Rapids, MI, United States; ^2^Neuroscience Program, Michigan State University, East Lansing, MI, United States; ^3^Mercy Health Hauenstein Neuroscience Center, Grand Rapids, MI, United States

**Keywords:** tau, axon, tauopathy, somatodendritic, microtubule-associated protein, rat, monkey, hippocampus

## Abstract

Tau is a microtubule-associated protein for which the physiological functions remain a topic of vigorous investigation. Additionally, tau is a central player in the pathogenesis of several diseases such as Alzheimer’s disease and several frontotemporal dementias. A critical variable to understanding tau in physiological and disease contexts is its normal localization within cells of the adult CNS. Tau is often described as an axon-specific (or enriched) and neuron-specific protein with little to no expression in glial cells, all of which are untrue. Understanding normal tau distribution also impacts interpretation of experimental results and hypotheses regarding its role in disease. Thus, we set out to help clarify the normal localization of tau in the adult CNS of middle-aged rats and rhesus macaque using the hippocampus as a representative brain structure. The physiological concentration of tau in the rat hippocampus was 6.6 μM and in white matter was 3.6 μM as determined by quantitative sandwich ELISAs. We evaluated the cellular localization of tau using multiple tau-specific antibodies with epitopes to different regions, including Tau1, Tau5, Tau7, R1, and two novel primate-specific antibodies NT9 and NT15. In the rat and monkey, tau was localized within the somatodendritic and axonal compartments, as well as a subset of neuronal nuclei. Semi-quantitative fluorescence intensity measurements revealed that depending on the specific reagent used the somatodendritic tau is relatively equal to, higher than, or lower than axonal tau, highlighting differential labeling of tau with various antibodies despite its distribution throughout the neuron. Tau was strongly expressed in mature oligodendrocytes and displayed little to no expression in oligodendrocyte precursor cells, astrocytes or microglia. Collectively, the data indicate tau is ∼3 – 7 μM under physiological conditions, is not specifically enriched in axons, and is normally found in both neurons and mature oligodendrocytes in the adult CNS. The full landscape of tau distribution is not revealed by all antibodies suggesting availability of the epitopes is different within specific neuronal compartments. These findings set the stage for better understanding normal tau distributions and interpreting data regarding the presence of tau in different compartments or cell types within disease conditions.

## Introduction

Tau was originally co-purified with microtubules from brain and was subsequently labeled as a microtubule-associated protein that primarily plays a role in stabilizing microtubules (Weingarten et al., 1975; Witman et al., 1976; Cleveland et al., 1977a, b). However, the functional repertoire of tau remains a matter of investigation and it appears to do much more than simply stabilize microtubules. In fact, evidence suggests tau is more likely involved in regulating microtubule dynamics (not stabilization), modulation of signaling pathways involved in axonal transport, and potentially additional roles including synaptic and nuclear functions, among others (Wang and Mandelkow, 2016; Combs et al., 2019; Mueller et al., 2021). The interest in better understanding tau function and dysfunction has steadily increased since the 1980’s when it was identified as the primary constituent of the pathological inclusions in Alzheimer’s disease (AD), known as neurofibrillary tangles (Brion et al., 1985; Delacourte and Défossez, 1986; Grundke-Iqbal et al., 1986a, b; Ihara et al., 1986; Kosik et al., 1986; Nukina and Ihara, 1986; Wolozin et al., 1986; Wood et al., 1986; Dickson et al., 1987). In fact, over the past decade or so there was a robust increase in studies on tau under normal and pathological conditions. Thus, it is critical that the field move forward with a clear understanding of basic biological characteristics of tau protein in the brain.

One important aspect of tau protein biology is its normal pattern of localization within neurons and glial cells of the adult brain. While a large body of literature exists regarding the pathological accumulation of tau in human diseases, there is much less work describing the normal distribution of tau in the CNS. A current dogma within the field is that tau is primarily a neuronal protein specifically localized and highly enriched in axons. Moreover, it is commonly thought to exist in negligible levels in other neuronal compartments and non-neuronal cells. The first description of tau as an “neuron- and axon-specific protein” was based upon one of the first monoclonal tau antibodies called Tau1 that was developed by the Binder group (Binder et al., 1985). The initial set of papers describing tau distribution in the CNS using Tau1 found no evidence that tau was located within the somatodendritic compartment of neurons or within glial cells (Binder et al., 1985, 1986; Busciglio et al., 1987; Dotti et al., 1987). As Tau1 was used in further studies using AD brain tissue, it became clear that the tau proteins in the pathological inclusions were phosphorylated and that removing phosphorylation dramatically increased the reactivity of pathology with Tau1 in disease tissue (Grundke-Iqbal et al., 1986b; Wood et al., 1986). With the new understanding that Tau1 reactivity was blocked by phosphorylation, the Binder group re-evaluated the physiological distribution of tau in the CNS (Papasozomenos and Binder, 1987). In these follow-up experiments, they clearly demonstrated that tau was in fact localized throughout the somatodendritic compartment of neurons and was present in glial cells, which they concluded were likely astrocytes and perineuronal glia (Papasozomenos and Binder, 1987). These results support the apparent fact that under physiological conditions the somatodendritic and glial tau is largely phosphorylated at the Tau1 site, while axonal tau is not. Over ∼30 years, a relatively small number of papers using various tau antibodies also highlighted the presence of tau within the somatodendritic and nuclear compartments of neurons and within glial cells (primarily interfascicular and perineuronal oligodendrocytes) (Migheli et al., 1988; Loomis et al., 1990; Brady et al., 1995; LoPresti et al., 1995; Irving et al., 1996; Thurston et al., 1996; Tashiro et al., 1997; Thurston et al., 1997; Gartner et al., 1998; Takahashi et al., 2000; Bullmann et al., 2009; Kubo et al., 2019a).

Despite the corrective conclusions published by the Binder group in the 80’s (Papasozomenos and Binder, 1987) and other studies, the ideas that the normal distribution of tau is neuron-specific and axon-specific are continually repeated in the literature and are the basis for formulating interpretations of experimental results. Interestingly, one contributing factor to the confusion could be the fact that tau is a highly flexible protein (i.e., a member of the so-called intrinsically disordered protein family) that likely undergoes dynamic regulation leading to the display of certain epitopes differentially within various cellular compartments or within cell populations under physiological conditions. This creates a situation in which specific antibodies will either label or not label particular pools of tau in the CNS despite its more ubiquitous localization. In addition, the differential distribution patterns revealed by Tau1 when it is used with or without dephosphorylation highlight the impact compartment-specific regulation of tau modifications could have on accurately identifying its normal distribution within cells.

Thus, we set out to further clarify the localization of tau within neurons and glia of the adult CNS under physiological conditions using multiple rigorously validated tau-specific monoclonal antibodies with epitopes to different regions of the tau protein. Using rat and rhesus macaque brain sections we show clear evidence that tau is localized within the somatodendritic and axonal compartments, as well as the nuclei of some neurons. Importantly, we demonstrate that some reagents are ineffective at labeling somatodendritic tau, despite its presence, while others label this pool of tau well. Among the antibodies that label somatodendritic tau effectively, we found equal or higher levels of somatodendritic tau when compared to axonal tau. These data highlight the ability of tau antibodies with various epitopes to differentially label tau in specific compartments despite the fact that it is normally located throughout the neuron and suggest that the availability of the epitopes is different within the neuronal compartments. Moreover, tau is strongly expressed in mature oligodendrocytes, but interestingly little to no expression was observed in oligodendrocyte precursor cells, astrocytes and microglia. These findings set the stage for better understanding distributions of tau under physiological conditions as well as interpreting data regarding the presence of tau in different compartments or cell types within disease conditions.

## Materials and Methods

### Animals

#### Mice

Adult female tau knock-out mice (NT9 antibody, B6.129X1-Mapttm1Hnd/J; Jackson Labs, 007251) or Balb/c mice (NT15 antibody, Jackson Labs, 000651) were used to generate tau antibodies (see below). Mice were housed with a 12 h light/dark cycle with food and water provided *ad libitum*. Wild-type C57/BL6J (Jackson Labs, 000664) and tau knockout mice also were used for positive and negative controls (cross-reactivity with non-tau proteins), respectively, in immunohistochemical staining and immunoblotting. Mice were given an overdose of Fatal Plus solution (≥100 mg/kg) and either transcardially perfused with 0.9% saline containing heparin (10,000 units/L) followed by 4% paraformaldehyde for histology or were perfused with only 0.9% saline/heparin for fresh tissue tau biochemistry before extracting the brains for processing. These studies were conducted in compliance with federal, state and institutional guidelines and approved by the Michigan State University Institutional Animal Care and Use Committee.

#### Rat

Naïve 14-month-old (i.e., middle-aged), male Fischer 344 rats (*n* = 4 for histology and *n* = 4 for tau biochemistry) were obtained through the National Institute on Aging rodent colony. Rats were housed 2 per cage in a room with a 12 h light/dark cycle and provided food and water *ad libitum*. Animals were given an overdose of Fatal Plus solution (≥100 mg/kg) and either transcardially perfused with 0.9% saline containing heparin (10,000 units/L) followed by 4% paraformaldehyde for histology or were not perfused for fresh tissue tau biochemistry before extracting the brains for processing. All work was performed at the AAALAC accredited Van Andel Research Institute vivarium with specific approval from the Michigan State Institutional Animal Care and Use Committee.

#### Monkey

Naïve male (*n* = 4) rhesus monkeys (Macaca mulatta) 14-16 years-old (i.e., middle-aged) were used. All animals were born in captivity, and ages were based on birth records. Based on gray and white matter changes in the brain, rhesus monkeys age at a ratio of 3:1 years compared with humans (Andersen et al., 1999). Therefore, our animals represent humans aged 42–48 years. Animals were individually housed and kept on a 12-h light/dark cycle. Animals were given an overdose of pentobarbital (50 mg/kg, i.v.) prior to transcardial perfusion with 0.9% saline. Then the brains were removed and the portion of the brain caudal to the anterior commissure was immersion fixed in 4% paraformaldehyde for 1 week followed by equilibration into 30% sucrose for ∼1–2 weeks. Tissue sections (40-μm thick) were cut in the coronal plane using a freezing-stage sliding knife microtome and stored in cryoprotectant solution at −20°C until processed for immunostaining. All applicable laws, regulations, and guidelines from the National Institutes of Health, United States Public Health Service Guide for the Care and Use of Laboratory Animals were followed. The animal use was approved by the Institutional Animal Care and Use Committees at Rush University Medical Center and the Biological Research Laboratory at the University of Illinois Chicago.

### Tau Sequence Alignments

**FIGURE 1:**
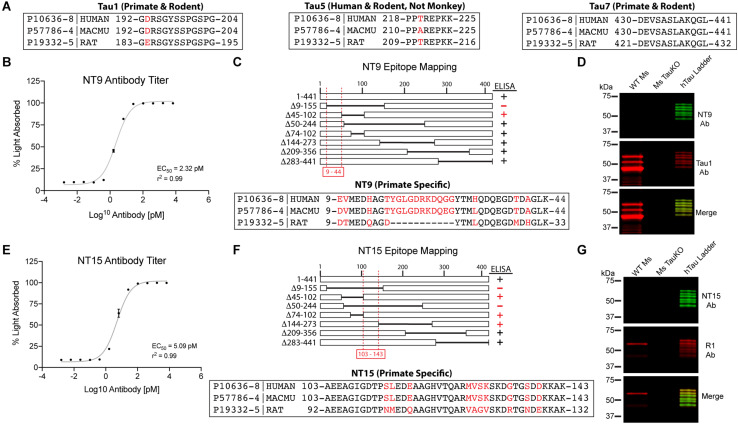
Established and novel anti-tau antibodies used in this study. **(A)** Amino acid sequence alignments for human (Uniprot ID P10636-8), rhesus macaque (Uniprot ID P57786-4), and rat (Uniprot ID P19332-5) tau proteins at the epitopes of Tau1 (IgG2a, aa192-204), Tau5 epitope (IgG1, aa 218-225), and Tau7 (IgG1, aa430-441) antibodies. Red residues indicate positions of species differences. **(B–D)** Characterization of the NT9 anti-tau antibody. **(B)** ELISA titers show that NT9 is sensitive with an EC_50_ value of 2.32 pM (non-linear sigmoidal curve fit, *r*^2^ = 0.99, *n* = 3, data represent mean ± SD). **(C)** ELISAs with full-length human tau protein and a series of overlapping deletion constructs (Δ9-155, Δ45-102, Δ50-244, Δ74-102, Δ144-273, Δ209-356, and Δ283-441). The loss of reactivity for Δ9-155 proteins, and positive reactivity with Δ45-102 protein places the epitope between amino acids 9-44 in human tau. The epitope alignment between human, monkey and rat is shown. **(D)** Brain lysates from wild-type mouse (WT Ms) and tau knockout mouse (Ms TauKO) brains were dephosphorylated (alkaline phosphatase treatment) and immunoblotted with NT9 (green) and Tau1 (red). A recombinant human tau (all 6 isoforms) ladder was used as a positive control, and NT9 reacted well with all 6 human tau isoforms. NT9 does not react with mouse tau and does not display non-specific bands in the Ms TauKO samples, showing that NT9 is primate-specific (human tau used) and tau-specific. **(E–G)** Characterization of the NT15 anti-tau antibody. **(E)** ELISA titers show that NT15 is sensitive with an EC_50_ value of 5.09 pM (non-linear sigmoidal curve fit, *r*^2^ = 0.99, *n* = 3, data represent mean ± SD). **(F)** ELISAs were performed using full-length tau and a series of deletion constructs (as above). The loss of reactivity for Δ9-155 and Δ50-244 proteins, and positive reactivity with Δ45-102, Δ74-102 and Δ144-273 places the epitope of NT15 between amino acids 103-143 in human tau. The epitope alignment between human, monkey and rat is shown. **(G)** WT Ms and Ms TauKO brain lysates and human tau ladder blots were probed with NT15 (green) and R1 (red). NT15 reacts well with all 6 human tau isoforms but does not react with mouse tau and does not display non-specific bands in the Ms TauKO samples, showing that NT15 is primate-specific (human tau used) and tau-specific.

The primary sequence of primates (including non-human primates and human primates) diverges most significantly from all other species in the extreme amino terminal region of the protein. We used the data from Uniprot^1^ to align primary sequences of the longest adult CNS isoform of tau (2N4R or hT40) from humans (ID# P10636-8, 441 amino acids), rhesus macaque monkey (ID# P57786-4, 441 amino acids), and rat (ID# P19332-5, 432 amino acids). The align function was utilized to produce the sequence alignments reported (Figure 1 and Supplementary Figure 1A).

### Generation and Characterization of Novel Anti-tau Antibodies, NT9 and NT15

We characterized two new N-terminal antibodies, referred to as NT9 (AB_2848138) and NT15 (AB_2848139), using techniques previously described in detail (Binder et al., 1985; Grabinski and Kanaan, 2016). Mice were immunized with either recombinant human 3R isoform tau protein aggregates (NT9) produced with arachidonic acid as described (Combs et al., 2017) or monomeric recombinant human 4R isoform tau proteins suspended in Freund’s adjuvant as described (Grabinski and Kanaan, 2016). Antibody hybridomas (fusion with SP2/o myeloma cells) were generated and screened using methods similar to those described previously by our group (Grabinski and Kanaan, 2016). Cultures were screened for reactivity against recombinant tau protein by indirect ELISAs. Cultures that were positive (absorbance > 1.0) were expanded and subcloned at least three times and confirmed for stability following freeze/thaw cycles. Antibody isotypes were determined using the IsoStrip Mouse Monoclonal Antibody Isotyping kit (11493027001, Roche). Once the final clones were verified as clean (i.e., mycoplasma negative) and stable, antibodies were purified and dialyzed into antibody storage buffer (10 mM HEPES, 500 mM NaCl, 50% glycerol) as described (Grabinski and Kanaan, 2016). Concentrations were measured using A280 (extinction coefficient 13.7) and antibodies were adjusted to 1mg/ml with antibody storage buffer, aliquoted, and stored at −80°C. Antibodies were characterized using ELISA titer assays, epitope mapping ELISAs and immunoblotting with wild-type mouse brain lysates, mouse tau knock-out brain lysates and a recombinant human tau isoform ladder (positive control).

### Indirect ELISAs

Indirect ELISAs were performed to determine the binding affinity, epitope and specificity of the NT9 and NT15 monoclonal antibodies as previously described (Grabinski and Kanaan, 2016). For the antibody titer ELISAs, 50 μl of recombinant human 4R tau protein (2N4R, 2 ng/μl) in borate saline solution (100 mM boric acid, 25 mM sodium tetraborate decahydrate, 75 mM NaCl, 250 μM thimerosal) was used to coat each well (Corning, 3590) for 1 h. Washing steps were done with ELISA wash solution (100 mM boric acid, 25 mM sodium tetraborate decahydrate, 75 mM NaCl, 250 μM thimerosal, 0.4% BSA and 0.1% tween-20, pH 9.0) (200 μl per well). Wells were blocked with 5% non-fat dry milk made in ELISA wash solution for 1 h. Purified NT9 and NT15 antibodies were diluted in blocking buffer at a range from 1:1,000 (6670 pM) to 1:4,194,304,000 (0.0016 pM) and incubated for 2 h. Goat anti-mouse IgG HRP conjugated antibody (Jackson ImmunoResearch, 115-035-003, AB_10015289) was added at 1:5,000 dilution for 1 h. Reactivity was detected by adding 3,3′,5,5′ tetramethylbenzidine substrate (Sigma, T0440) and incubating for 15 min. Reactions were quenched with 50 μl 3.6% H_2_SO_4_ and absorbance read at 450 nm. Absorbance (A) is not linear [i.e., A = Log_10_(1/transmittance)], thus, the absorbance data were converted to percent absorbed light (a linear scale) using the following equation %A = (1 – 10^–*x*^)^∗^100, where x is absorbance. Graphs and EC_50_ concentrations were produced using GraphPad Prism software. Epitope mapping indirect ELISAs were performed as described above except the plates were coated with a series of overlapping deletion human tau protein constructs (Δ9-155, Δ45-102, Δ50-244, Δ74-102, Δ144-273, Δ209-356 or Δ283-441; numbering based on human 2N4R isoform of 441 amino acids). Negative signal was set ≤11% light absorbed (i.e., close to background levels) and all positive signals were >63% light absorbed.

### Quantitative Sandwich ELISA of Physiological Tau Levels

Brains were immediately removed from unperfused naïve rats (*n* = 4), briefly rinsed in ice-cold 0.9% saline to remove excess blood and then the surface was gently blotted dry. Bilateral hippocampus (HP, represents gray matter-enriched sample) or pooled white matter (WM) comprised of bilateral corpus callosum, fimbria and cerebellar peduncles (represents WM-enriched samples) were collected and frozen immediately on dry ice. The surrounding white matter (fimbria/alveus) was excluded as much as possible from the HP samples, while the surrounding gray matter was excluded from the WM samples. Samples were processed using the following procedures to carefully track sample weights and volumes in an attempt to accurately account for sample dilutions. Tissue weight was determined by subtracting pre-sample tube weights from combined weight of brain tissue and the tube. Ice-cold lysis buffer consisting of 20mM Tris, pH 7.5, 0.5 mM DTT, 0.15M NaCl, and 0.5% Triton X-100 supplemented with Complete Protease Inhibitor Cocktail Tablets (Roche, 04693116001) was added to bring each sample to approximately 300 μl, the tubes were weighed again to obtain an accurate measurement of lysis buffer volume added to the tissue (1 μl buffer = 1 mg weight), and then samples were sonicated to homogenize the tissue (Ultrasonic XL-2000 series sonicator). The samples were then measured by pipette to more accurately determine final sample volumes. Starting tissue volume was determined by subtracting the known buffer volume added from the measured final sample volume after sonication. Samples were centrifuged at 18,000 × *g* for 5 min at 4°C to remove insoluble material and cellular debris and the supernatants containing total detergent soluble proteins were used for further analysis. Total soluble protein concentrations of final lysates were determined using Bradford protein assays (BioRad, 5000006). This process allowed for back calculations of brain weight to volume (g/ml), tau molarity (μM), tau concentration (mg/ml), and ratio of tau (μg) to soluble total protein (mg).

The detergent soluble protein lysates were used in a quantitative sandwich ELISA to determine the concentration of tau proteins using methods similar to those previously described (Kanaan et al., 2015a; Combs et al., 2016; Tiernan et al., 2016). Briefly, high-binding capacity 96 well plates (Corning, #3590) were coated with the Tau7 antibody (50 μl/well at 2 ng/μl in borate saline; Kanaan Lab, AB_2721195) as the capture antibody and incubated for 1 h at room temperature (Horowitz et al., 2006). Wells were washed (wash buffer: 100 mM boric acid, 25 mM sodium borate decahydrate, 75 mM NaCl, 247 uM thimerosal, 60 uM bovine serum albumin, 0.05% Tween-20, pH 9.0) and then blocked using 5% milk in wash buffer for 1 h at room temperature. Brain lysates were adjusted to 100 ng/μl (5 μg total protein/well) in TBS (50 mM Tris, 0.15 M NaCl, pH 7.4) and incubated for 1.5 h at room temperature. Recombinant mouse tau (0 – 20 nM, diluted in TBS) was used as a standard and there is 100% homology between rat and mouse tau at Tau5 and Tau7 epitopes (Supplementary Figure 1). Wells were washed and then the captured tau protein was detected with biotinylated Tau5 antibody (1:1000 in TBS; Kanaan lab, AB_2721194, LoPresti et al., 1995; Carmel et al., 1996) for 1.5 h at room temperature. Tau5 biotinylation was performed using EZ-Link NHS-PEG4-Biotin (Thermo Fisher Scientific, A39259). Next, horseradish peroxidase conjugated streptavidin (Jackson Immuno Research, 016-030-084) diluted 1:5000 in TBS was incubated for 1 h at room temperature. Signal was detected by incubating 3,3′,5,5′-tetramethylbenzidine substrate (50 μl/well; Sigma, T8665) for 20 min and quenched using 3.6% H_2_SO_4_ (50 μl/well). Absorbance at 450 nm was read on a Molecular Diagnostics Spectra Max Imager Plus plate reader. The standard curve of recombinant mouse tau was fit to a non-linear sigmoidal curve (*r*^2^ = 0.99) and used to interpolate the concentration of tau in the samples from the HP and WM (comprised of the corpus callosum, fimbria, and cerebellar peduncles).

### Western Blotting

Monoclonal antibodies NT9 and NT15 were validated with a human tau ladder containing all six isoforms (rPeptide, T-1007-2) and wild-type and tau knock-out mouse brain lysates. Cortical brain samples were homogenized in lysis buffer [20 mM Tris pH 7.5, 0.5 mM DTT, 300 mM NaCl, 0.5% Triton-X100 (Tx)] supplemented with protease (10 μg/ml Aprotinin, Pepstatin, Leupeptin, Bestatin, 500 mM PMSF) and phosphatase inhibitors (1 mM Tetra-sodium pyrophosphate decahydrate,10 mM beta-glycerophosphate disodium salt pentahydrate, 1 mM Sodium orthovanadate, 10 mM sodium fluoride), sonicated and centrifuged at 12,000 × *g* for 10 min and supernatant collected. Protein concentrations were determined using the Bradford Protein Assay (BioRad, 500-0006) according to manufacturer’s instructions. Samples were dephosphorylated overnight at 37°C using FastAP Thermosensitive Alkaline Phosphatase (Fermentas, EF0651) prior to bringing each sample concentration to 1.67 mg/ml with Laemmli sample buffer and heating at 98°C for 5 min. Human tau ladder was loaded at 5 μl (25 ng each isoform) and dephosphorylated brain lysates were loaded at 50 μg/lane and separated using SDS-PAGE on 4-20% Tris-HCl Criterion gels (BioRad, 567-1093). Proteins were transferred onto BioTrace nitrocellulose membrane (VWR, 27376-991) and blocked for 1 h with 2% non-fat dry milk in TBS (500 mM Tris, 150 mM NaCl, pH 7.4) prior to incubation in primary antibodies NT9 (1:200,000; mouse IgG1; AB_2848138) and Tau1 (1:1,000,000; mouse IgG2a, Kanaan lab, AB_2721197, Binder et al., 1985; Grundke-Iqbal et al., 1986b; Wood et al., 1986; Papasozomenos and Binder, 1987; Liu et al., 1993; Carmel et al., 1996) or NT15 (1:90,000; mouse IgG1; AB_2848139) and R1 (1:100,000; rabbit polyclonal, Kanaan lab, AB_2832929, Berry et al., 2004) overnight at 4°C. The NT9-Tau1 blot was incubated for 1 h with secondary antibodies IRDye 680LT goat anti-mouse IgG1 (1:20,000; LiCor, 926-68050; AB_2783642) and IRDye 800CW goat anti-mouse IgG2a (1:20,000; LiCor, 926-32351; AB_2782998), while the NT15-R1 blot was incubated for 1 h with IRDye 680LT goat anti-mouse IgG1 (1:20,000; LiCor, 926-68050; AB_2783642), and IRDye 800CW goat anti-rabbit (1:20,000; LiCor, 926-32211; AB_621843). Blots were imaged on a Licor Odyssey system with ImageStudio software (v5.2.5, LiCor Biosciences). Control membranes were run using the protocol outlined above excluding primary antibodies to rule out endogenous mouse immunoglobulin detection in brain lysate samples.

We analyzed the relative abundance of multiple protein markers to further validate the enrichment of somatodendritic components in the HP and axonal component enrichment in the WM samples from rat brains (*n* = 4). To dephosphorylate proteins for Tau1 immunoblotting, the samples were incubated at 37°C for 2 h with 100 U Thermosensitive Alkaline Phosphatase in 1x FastAP buffer (Thermo Fisher Scientific, EF0651) supplemented with protease inhibitor cocktail (Roche, 11836170001) prior to preparation for SDS-PAGE. Standard sample preparation was used for all other blots. Samples were adjusted to 0.67 μg/μl in 6x Laemmli sample buffer, heated at 98°C for 5 min and then 20 μg total protein/lane was separated using SDS-PAGE on 4-20% Tris-HCl Criterion gels and transferred to nitrocellulose (as above). Membranes were probed with Revert 700 Total Protein Stain Kit (Licor, 926-11010) per the manufacturer’s instructions to assess total proteins and for utilization in signal normalization. The membranes were destained (per instructions), blocked and probed with primary antibodies overnight at 4°C. Primary antibodies included Tau1 antibody (1:100,000), AP14 microtubule associated protein 2 (MAP2) antibody (1:500; a somatodendritic marker; AB_2832940, Caceres et al., 1984; Binder et al., 1986; Tucker et al., 1988; Kalcheva et al., 1994), post-synaptic density-95 antibody (PSD-95, 1:1,000; a post-synaptic protein enriched in somatodendritic compartment; Cell Signaling Technologies, 3450), Tuj1 βIII-tubulin antibody (1:10,000; a neuron-specific tubulin isoform in somatodendritic and axonal compartments; Caccamo et al., 1989), SMI-312 neurofilament antibody (1:1,000; a phospho-neurofilament axon-specific marker, Biolegend, 837904), and myelin basic protein antibody (MBP, 1:1,000; an oligodendrocyte marker enriched in WM tracts; Cell Signaling Technologies, 78896). Membranes were rinsed after primary antibody incubations and then incubated with appropriate secondary antibody (diluted 1:20,000; reagent details above) for 1 h at room temperature. PSD-95 and MBP were labeled with goat anti-rabbit 800 antibody, Tau1 and Tuj1 were labeled with goat anti-mouse IgG2a 800 antibody, AP14 was labeled with goat anti-mouse IgG1 680 antibody, and SMI-312 was labeled with goat anti-mouse IgG (H+L) 680 antibody. Membranes were rinsed and imaged as above. Quantification of immunoreactive bands was performed using Licor Image Studio Software (v5.2.5). Signal intensity values for each target protein bands were normalized to Revert signal obtained in a region between ∼75 and 37 kDa (this region of the blot showed similar band patterns between HP and WM). Normalized signal intensity values were used for target protein enrichment comparisons between in HP and WM samples.

### Immunohistochemistry

Immunohistochemistry was performed on free-floating rat and monkey brain sections (40 μM thick) following established protocols (Kanaan et al., 2007; Combs et al., 2016). For Tau1 staining, sections were either untreated or dephosphorylated overnight at 37°C using FastAP Thermosensitive Alkaline Phosphatase (1 U/μl; Fermentas, EF0651) diluted in 1:100 in buffer prior to proceeding with staining. Tissue was washed in TBS with 0.5% TritonX-100 (TBS-Tx), endogenous peroxidase activity quenched in 3% H_2_O_2_ for 1 h and then blocked in 10% goat serum/2% BSA/0.4% Tx for 1 h prior to incubation with primary antibodies Tau1 (1:60,000), Tau7 (1:50,000; mouse IgG1; AB_2721195), NT15 (1:20,000), NT9 (1:30,000), Tau5 (1:20,000; AB_2721194), or R1 (1:6,000) overnight at 4°C (all diluted in 2% goat serum TBS-Tx), followed by goat anti-mouse biotinylated secondary antibody at 1:500 diluted in 2% goat serum TBS-Tx (Jackson ImmunoResearch, 115-065-166; AB_2338569) and then ABC Elite solution (Vector Labs, PK-6100). The tissue was developed using 3,3′-diaminobenzidine (Sigma, D5637) at 0.5 mg/ml in TBS-Tx with 0.003% H_2_O_2_ for 8 min. Sections were rinsed, mounted on microscope slides and processed through ethanol and xylenes before coverslipping with Cytoseal-60 (Thermo, 831016).

The Tau1, R1, Tau5, and Tau7 antibodies were further validated using wild-type mouse and tau knockout mouse tissue sections. The tau knockout sections serve as a negative control and confirm whether the procedures used produce non-specific background staining (Supplementary Figure 2). As additional negative controls, monkey sections were stained using Tau5 (does not react with monkey tau) and rat sections were stained using NT9 and NT15 (do not react with rat tau, primate-specific antibodies) (Supplementary Figure 2). Free-floating fixed tissue sections from the inferior temporal gyrus of a severe AD (Braak V–VI) case from the Brain Bank of the Cognitive Neurology and Alzheimer’s Disease Center at Northwestern University were used to confirm the effectiveness of the tissue dephosphorylation procedure (i.e., treatment with phosphatase) and to establish whether phosphorylation impacted, R1, Tau7, NT9, and NT15 immunostaining (Supplementary Figure 3). Since Tau7 staining patterns in adult rat brain sections resemble Tau1 staining prior to dephosphorylation (i.e., no somatodendritic labeling), we also included a phosphatase treatment test with Tau7 in normal rat sections. Finally, monkey sections were also included in the phosphatase treatment test to confirm whether NT9 and NT15 were impacted by the phosphorylation status of tau in normal adult monkey brains.

Images were acquired at 20x magnification with a Nikon Eclipse 90i microscope, a Nikon DS-Ri1 camera, and Nikon Elements AR software (Nikon Instruments Inc., Melville, NY, United States). All images for a stain series were acquired using identical microscope parameters (magnification, light source intensity, exposure time, and contrast). The images were prepared for publication using Adobe Photoshop and Illustrator.

### Multilabel Immunofluorescence

Immunofluorescence staining was performed on rat and monkey brain tissue using previously established protocols (Kanaan et al., 2007; Combs et al., 2016). For Tau1 staining, tissue sections were dephosphorylated overnight at 37°C as above. Sections were washed in TBS-Tx and blocked with 10% goat serum/2% BSA/0.4% Tx for 1 h at room temperature. A set of rat tissue was stained with Tau1 (IgG2a, 1:6,000), Tau7 (IgG1, 1:5,000), and MAP2 (rabbit IgG, 1:300; Cell Signaling Technology, 8707) primary antibodies overnight at 4°C, followed by Alexa Fluor goat anti-mouse IgG2a 568 (Thermo Fisher Scientific, A21134; AB_2535773), goat anti-mouse IgG1 647 (Thermo Fisher Scientific, A21240; AB_2535809), and goat anti-rabbit 488 (Thermo Fisher Scientific, A32731; AB_2633280) secondary antibodies (all at 1:500) for 2 h to assess cellular compartment distribution of tau. A second set of rat tissue was incubated with Tau5 (IgG1, 1:2,000) and R1 (rabbit IgG, 1:600) overnight at 4°C, followed by the Alexa Fluor goat anti-mouse IgG1 647 (Thermo Fisher Scientific, A21240) and Alexa Fluor goat anti-rabbit 568 (Thermo Fisher Scientific, A11011; AB_143157) secondary antibodies (all at 1:500) for 2 h to assess the co-localization between the tau antibodies in rat brains. A third set of rat tissue was incubated in primary antibodies Tau1 (IgG2a, 1:6,000), oligodendrocyte lineage transcription factor 2 (Olig2; rabbit monoclonal; 1:50; Abcam, 109186; AB_10861310) and myelin basic protein (MBP; IgG2b; 1:100; Cell Signaling Technologies, 83683) overnight at 4°C, followed by incubation in Alexa Fluor goat anti-mouse IgG2a 488 (Thermo Fisher Scientific, A21131; AB_2535771), goat anti-rabbit 568 (Thermo Fisher Scientific, A11011), and goat anti-mouse IgG2b 647 (Thermo Fisher Scientific, A21242; AB_2535811) secondary antibodies (all at 1:500) for 2 h to assess whether tau-positive glia were mature oligodendrocytes (MBP and Olig2). A fourth set of rat tissue was incubated in primary antibodies Tau1 (IgG2a, 1:6,000), Olig2 (Abcam, 109186), and neural/glial antigen 2 (NG2; IgG1; 1:800; Abcam, ab50009; AB_881569) overnight at 4°C, followed by incubation in Alexa Fluor goat anti-mouse IgG2a 488 (Thermo Fisher Scientific, A21131; AB_2535771), goat anti-rabbit 568 (Thermo Fisher Scientific, A11011), and goat anti-mouse IgG1 647 (Thermo Fisher Scientific, A21240) secondary antibodies (all at 1:500) for 2 h to assess whether tau-positive glia were oligodendrocyte precursor cells (NG2 and Olig2). A fifth set of rat tissue was incubated in primary antibodies Tau1 (IgG2a, 1:6,000), ionized calcium-binding adapter molecule 1 (IbaI; rabbit polyclonal, 1:1000; Wako, 019-19741; AB_839504) and glial fibrillary acidic protein (GFAP; IgG1; 1:400; Sigma, G3893; AB_477010) overnight at 4°C, followed by incubation in Alexa Fluor goat anti-mouse IgG2a 488 (Thermo Fisher Scientific, A21131), goat anti-rabbit 568 (Thermo Fisher Scientific, A11011), and goat anti-mouse IgG1 647 (Thermo Fisher Scientific, A21240) secondary antibodies (all at 1:500) for 2 h to assess whether tau-positive glia were microglia (IbaI) or astrocytes (GFAP). All primary and secondary antibodies were diluted in 2% goat serum TBS-Tx and between each step above sections were washed 4 times for 5 min with TBS-Tx.

A set of monkey brain sections was incubated with NT15 (IgG1, 1:2,000) and R1 (rabbit IgG, 1:600) overnight at 4°C, followed by Alexa Fluor goat anti-mouse IgG1 647 (Thermo Fisher Scientific, A21240) and Alexa Fluor goat anti-rabbit 568 (Thermo Fisher Scientific, A11011) secondary antibodies diluted 1:500 in 2% goat serum TBS-Tx for 2 h. A second set of monkey tissue was incubated in primary antibodies NT15 (IgG1, 1:2,000) and MBP (IgG2b; 1:100; Thermo Fisher Scientific, MA1-10837; AB_1077025) overnight at 4°C, followed by incubation in Alexa Fluor goat anti-mouse IgG1 488 (Thermo Fisher Scientific, A21121; AB_2535764) and goat anti-mouse IgG2b 647 (Thermo Fisher Scientific, A21242) secondary antibodies (all at 1:500) for 2 h to assess whether tau-positive glia were mature oligodendrocytes (MBP). A third set of monkey tissue was incubated in primary antibodies Tau1 (IgG2a, 1:6,000), IbaI (1:1000; Wako, 019-19741), and GFAP (1:400; Sigma, G3893) overnight at 4°C, followed by incubation in Alexa Fluor goat anti-mouse IgG2a 488 (Thermo Fisher Scientific, A21131), goat anti-rabbit 568 (Thermo Fisher Scientific, A11011), and goat anti-mouse IgG1 647 (Thermo Fisher Scientific, A21240) secondary antibodies (all at 1:500) for 2 h to assess whether tau-positive glia were microglia (IbaI) or astrocytes (GFAP). All primary and secondary antibodies were diluted in 2% goat serum TBS-Tx and between each step above sections were washed 4 times for 5 min with TBS-Tx.

All tissue sections in the above sets of immunofluorescence stains were washed in TBS-Tx containing 4′,6-Diamindino-2-Phenylindole (DAPI) (0.5 μg/ml, D1306, Thermo) prior to mounting. Sections were mounted on microscope slides and stained with Sudan Black (20 mg/ml in 70% ethanol) (Thermo Fisher Scientific, BP109). Briefly, sections were dehydrated in 70% ethanol for 1 min, incubated in Sudan Black stain for 5 min, differentiated in 70% ethanol, rinsed in H_2_O two times for 3 min each and then coverslipped with Vectashield Hard Set Mounting Medium (Vector Labs, H1400). Control sections were prepared using the same methodology but without each primary antibody individually or without all of the primary antibodies (Supplementary Figures 2-5). Images were acquired and analyzed as described below.

### Confocal Imaging and Analysis

All confocal imaging was performed on a Nikon A1+ laser scanning confocal microscope system equipped with 488, 561, and 640 solid-state lasers, 40x (1.3 na) and 60x (1.4 na) objectives and Nikon Elements AR software. High magnification (60x) z-stack images (0.5 μm step size) were obtained for qualitatively determining co-localization within hippocampal regions in sections stained with either MAP2+Tau1+Tau7+DAPI (rat), Tau5+R1+DAPI (rat), Tau1+MBP+Olig2+DAPI (rat), Tau1+NG2+Olig2+DAPI (rat), Tau1+IbaI+GFAP+DAPI (rat), NT15+R1+DAPI (monkey), NT15+MBP+DAPI (monkey), Tau1+IbaI+GFAP+DAPI (monkey), or the indicated primary delete control stains (Supplementary Figures 4–11). Images displayed are maximum projections of 2-3 slices from a z-stack. For image analysis, z-stack images were acquired at 40x magnification with a 0.5 μm step size through 3 μm (7 slices total) of the regions of interest. Images of Tau1, Tau7, Tau5, or R1 stained hippocampal sections were used as described below for semi-quantitative signal intensity measurements. All images were prepared for publication using Adobe Photoshop and Illustrator. All images for each stain were acquired using identical microscope parameters (magnification, laser intensity, exposure time, contrast, scan mode and pin hole size).

Image stacks from the above sections were used to measure the relative fluorescence intensity of Tau1 or Tau7 (same sections) and Tau5 or R1 (same sections) signal in the alveus (Alv, axons of CA1 neurons), stratum oriens (SO, mixed population of axons and dendrites), and somata of individual CA1 neurons. Regional average intensity measurements of tau signal were used in the regions of the Alv and SO due to difficulty in distinguishing discrete specific structure. This was measured using six boxes (equally sized) placed throughout the region, avoiding structures such as blood vessels or positive interfascicular glia and taking intensity readings in each slice of the z-stack (total of 3 z-stack images per stain per animal × 7 z-slices/stack × 6 boxes per region produced 126 measures per animal). Neurons are discrete structures, thus, the cytoplasm was manually outlined to exclude the nuclei and measurements were made in a single z-plane where the nuclei were largest (i.e., representative of the center of the cell) and both nuclei and cytoplasm were definable (an average of 80 cells were analyzed per stain per animal). This approach provides a semi-quantitative measurement of relative differences in tau levels among individual cells and parenchymal/white matter areas. Respective antibody primary delete sections were used to obtain background fluorescence levels which were then subtracted from the signals obtained in stained sections. Background adjusted intensity levels were averaged for each animal and these data were used in statistical comparisons as outlined below.

### Statistical Analyses

The semi-quantitative immunofluorescence image analysis data were analyzed using a repeated measures one-way ANOVA (ROIs within animals are dependent variables) with Tukey’s *post hoc* analysis. The normalized signal intensity values from immunoblot analysis were compared between HP and WM using a paired *t*-test. Statistical significance was set at *p* ≤ 0.05. All data in graphs represent the mean ± SD. No data were excluded from analysis in this study.

## Results

### Tau Antibodies

A number of established (Tau1, Tau5, Tau7, and R1) and two novel primate-specific (NT9 and NT15, “primate” refers to human and monkey) tau antibodies were used to determine the normal distribution of tau in neurons and glia of the adult rat and rhesus macaque brain and whether antibodies with epitopes to different regions in tau would differentially reveal subcellular localization. All of the established anti-tau reagents were characterized in prior work and are high-affinity, high-specificity reagents. Tau1 is a mouse IgG2a that labels amino acids 192-204 (Figure 1A) and is sensitive to phosphorylation, requiring dephosphorylation of samples to effectively label all tau proteins (Binder et al., 1985; Grundke-Iqbal et al., 1986b; Wood et al., 1986; Papasozomenos and Binder, 1987; Liu et al., 1993; Carmel et al., 1996). Tau1 reacts with all 6 tau isoforms and there is 100% sequence homology in the Tau1 epitope when comparing rhesus macaque and human tau proteins, and rat is 92.3% homologous (aa 183-195 in rat) with a single difference of glutamic acid at position 193 (aspartic acid in primates; Figure 1A). The Tau5 antibody is a mouse IgG1 monoclonal antibody that was raised against full-length bovine tau (LoPresti et al., 1995; Carmel et al., 1996). This antibody was originally described to bind between residues 210-230 (Carmel et al., 1996), but more recent refinement suggests the epitope is residues 218-225 (Porzig et al., 2007). This epitope is 100% conserved between rats (aa 209-216 in rat) and humans, while rhesus macaques have a single residue difference of an alanine at position 220 instead of the threonine in rats and humans (Figure 1A). Surprisingly, Tau5 did not stain tau in monkey brain sections suggesting the single T-A difference is critical to Tau5 reactivity (Supplementary Figure 2C). The Tau7 antibody is a mouse IgG1 monoclonal reagent that labels the extreme C-terminus of tau between residues 430-441 (Horowitz et al., 2006). This area of tau has identical sequence homology between rat (aa 421-432 in rat), rhesus macaque and humans (Figure 1A). The R1 antibody is a rabbit polyclonal with epitopes throughout the tau protein, many of which are in the N-terminal half of the protein (Berry et al., 2004). Although defining specific epitopes within polyclonal antibodies is difficult, R1 is known to react with both primate and rodent tau protein.

The newly described NT9 and NT15 anti-tau antibodies were used to better capture distribution patterns in monkey using reagents with epitopes in additional regions within tau. The NT9 tau antibody is a mouse IgG1 monoclonal reagent and ELISA titers with full-length human tau protein indicate NT9 is a high-affinity reagent (EC_50_ = 2.32 pM; Figure 1B). The NT9 epitope was determined using overlapping deletion tau protein constructs (Δ9-155, Δ45-102, Δ50-244, Δ74-102, Δ144-273, Δ209-356 or Δ283-441) in ELISAs. The loss of reactivity against Δ9-155 protein, and positive reactivity with Δ45-102 protein (as well as all other deletion constructs) indicates that the epitope resides in the extreme N-terminus of tau and is between residues 9-44 of human tau (Figure 1C). The epitope alignment between human, monkey and rat is shown (Figure 1C). This area is relatively well conserved between humans and rhesus macaques (∼92% homology), while there are significant N-terminal differences between rat and primate tau (∼73% homology; aa 9-33 in rat; Figure 1C). Rat tau does not contain the primate sequence between tyrosine 18 and 29 as well as 6-7 other residue differences, which likely account for the lack of NT9 reactivity (Figure 1C). Immunoblotting of wild-type mouse and tau knockout mouse brain lysate was used to further characterize NT9 specificity (Figure 1D). NT9 reacted well with the recombinant human tau isoform ladder (i.e., positive control), but did not show reactivity with wild-type mouse (Tau1 was used to label mouse tau) confirming primate tau specificity and did not show non-specific bands in tau knockout mouse (similar to Tau1) indicating tau protein specificity (Figure 1D). Thus, the NT9 tau antibody is primate (i.e., human and monkey) tau-specific.

The NT15 tau antibody is a mouse IgG1 monoclonal reagent and ELISA titers with full-length human tau protein indicate NT15 is a high-affinity reagent (EC_50_ = 5.09 pM; Figure 1E). The NT15 epitope was determined using overlapping deletion tau protein construct ELISAs (as above). The loss of reactivity against Δ9-155 and Δ50-244 proteins, and positive reactivity with Δ45-102, Δ74-102 and Δ144-273 proteins (as well as all other deletion constructs) indicates that NT15 recognizes an N-terminal epitope between residues 103-143 in full-length human tau (Figure 1F). The alignment of this epitope shows 100% conservation between humans and rhesus macaques, while rat tau contains 10 different residues as well as 2 additional residues inserted at threonine 149 in primate tau (∼81% homology; aa 92-132 in rat; Figure 1F). Immunoblotting of the human tau isoform ladder, wild-type mouse and tau knockout mouse brain lysate show that NT15 reacted well with the recombinant human tau isoform ladder (i.e., positive control), but did not show reactivity with wild-type mouse confirming primate tau specificity (R1 was used to label mouse tau) and did not show non-specific bands in tau knockout mouse (similar to R1) indicating tau protein specificity (Figure 1G). Thus, the NT15 tau antibody is primate (i.e., human and monkey) tau-specific.

Tau antibodies were further validated for cross-reactivity with other proteins in immunohistochemical staining using wild-type and tau knockout mouse tissue. The Tau1 (with dephosphorylation), R1, Tau5, and Tau7 antibodies reacted with tau in cortical sections from wild-type mice, but none of them showed cross-reactivity in tau knockout mice indicating they are indeed specific to tau and that the immunohistochemical procedures are free from background artifacts (Supplementary Figures 2A–D). Furthermore, we show that Tau5 does not produce cross-reactivity in monkey hippocampal sections confirming the lack of cross-reactivity with other proteins and quality of the immunohistochemical procedure (Supplementary Figure 2C). Finally, we used rat sections to demonstrate the primate-specificity of NT9 and NT15 antibodies and that these new tau antibodies do not show non-specific cross-reactivity with other proteins. Neither NT9 nor NT15 showed reactivity in rat sections (Supplementary Figures 2E,F). These data demonstrate the specificity of the tau antibodies and lack of non-specific background staining with these reagents.

Next, we confirmed that R1, Tau7, NT9, and NT15 antibodies are not impacted by the phosphorylation status of tau proteins using temporal lobe cortical sections from a neuropathologically confirmed Alzheimer’s disease patient. Note that Tau5 is established as a phosphorylation independent antibody (Carmel et al., 1996). Sections from the inferior temporal gyrus were either untreated or pre-treated with phosphatase prior to immunohistochemical staining. We found that the patterns and extent of staining with R1, Tau7, NT9, and NT15 were unaffected by phosphatase treatment in AD pathology (Supplementary Figures 3B–E). This was in stark contrast to the robust increase in tau pathology detection observed with Tau1 immunostaining in phosphatase-treated sections compared to the untreated tissue, which also confirms the success of the dephosphorylation treatment procedure (Supplementary Figure 3A). We also tested whether the staining pattern of Tau7 in normal rat tissue was impacted by phosphorylation and found no change in Tau7 reactivity with or without dephosphorylation (Supplementary Figure 3C). This finding indicates that the lack of somatodendritic tau with Tau7 antibody is not likely due to phosphorylation in the epitope. Lastly, we included monkey sections that were either untreated or treated with phosphatase to establish if NT9 and NT15 are affected by phosphorylation in the normal monkey brain. There were no observable differences in the pattern and extent of labeling with either antibody in the HP (gray matter area) and alveus (white matter area) confirming they are not likely impacted by tau phosphorylation (Supplementary Figures 3D,E). These data demonstrate that R1, Tau7, NT9, and NT15 reactivity does not appear to be affected by tau phosphorylation that occurs in tissue.

### Physiological Tau Concentration in the Rat Hippocampus and White Matter

We set out to establish the physiological level of tau in the rat HP (representing gray matter-enriched brain samples) and WM from the corpus callosum, fimbria and cerebellar peduncles (representing WM-enriched brain samples) using quantitative sandwich ELISAs (Table 1 and Supplementary Figure 1). We determined that physiological soluble tau concentrations were 6.6 μM in the HP and 3.6 μM in the WM, which are equivalent to 0.30 and 0.16 mg/ml, respectively (Table 1). We used 41.3 kDa for molarity calculations, which represents a generalizable average molecular weight for tau isoforms (similar in humans, rats, and bovine). In addition, the original brain tissue weight/volume was calculated at ∼1 g/ml and total soluble protein was 120 – 130 mg/ml for the HP and WM. These calculations were used to extrapolate that there is 2.1 or 1.2 μg tau per mg of total soluble protein in the HP or WM, respectively (Table 1).

**TABLE 1:** Quantitative sandwich ELISA measurements of endogenous tau levels in rat hippocampus (HP) and white matter (WM).

Brain Region	Endogenous Tau (μM)	Endogenous Tau (mg/ml)	Brain wt/vol (g/ml)	Total Soluble Protein (mg/ml)	Tau per Total Soluble Protein (μg/mg)
**HP**	6.6 (±1.7)	0.30 (±0.09)	1.06 (±0.18)	130.8 (±9.0)	2.1 (±0.71)
**WM**	3.6 (±0.9)	0.16 (±0.04)	1.07 (±0.20)	120.7 (±11.0)	1.2 (±0.28)

*All data are group mean ± standard deviation (*n* = 4). Recombinant mouse tau used as a standard curve (0 – 20 nM) analyzed using a nonlinear sigmoidal curve fit (*r*^2^ = 0.99) to interpolate rat lysate samples concentrations. Tau molarity calculated using molecular weight of 41.3 kDa.*

**FIGURE 2:**
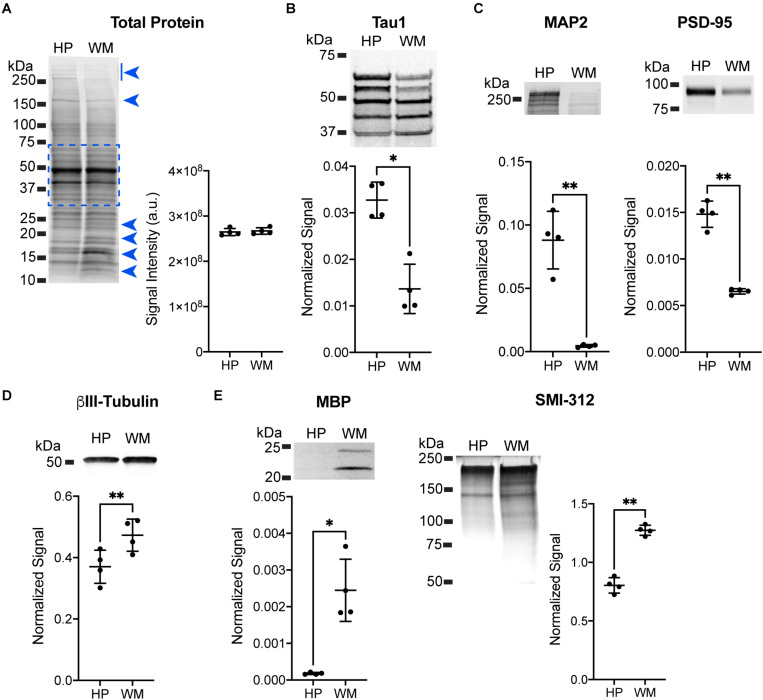
Immunoblotting confirmation of enrichment for somatodendritic components in hippocampal samples and axonal components in white matter samples. (**A**) Equal loading of hippocampal (HP) and white matter (WM) samples was confirmed using Revert protein stain reagent. Analysis of all bands between ∼75 and 37 kDa (indicated by dashed blue box, this area avoids clearly differentially distributed protein bands indicated by blue arrowheads) confirmed equal loading between HP and WM samples (representative blot shown, *p* = 0.73). (**B**) HP and WM samples were treated with phosphatase to dephosphorylate the sample prior to separation and immunoblotting with Tau1 antibody. The HP contained significantly greater tau levels compared to the WM (2.4-fold, ^∗^*p* = 0.163), with all bands except the lowest showing enrichment in the HP. (**C**) MAP2 and PSD-95 were used as known markers of the somatodendritic compartment to confirm enrichment in the HP sample. Indeed, there was a significant increase of 19.6-fold (^∗∗^*p* = 0.0046) and 2.3-fold (^∗∗^*p* = 0.0012), respectively, with each marker in the HP when compared to the WM. (**D**) βIII-tubulin was used as a neuron-specific tubulin isoform that is present in both somatodendritic and axonal compartments and is a major binding substrate for tau. βIII-tubulin signal was significantly increased (1.3-fold, ^∗∗^*p* = 0.0019) in WM when compared to the HP. (**E**) Myelin basic protein (MBP) and an axon-specific neurofilament marker (SMI-312) were used as markers of axons to confirm enrichment in WM sample. Both MBP and SMI-312 were significantly increased by 13.8-fold (^∗∗^*p* = 0.0136) and 1.6-fold (^∗∗^*p* = 0.0022), respectively, in the WM when compared to the HP.

Immunoblotting for specific protein targets was performed to further establish the enrichment of somatodendritic components in the HP and axonal components in the WM samples. All samples were adjusted to equal total protein levels in each lane and the Revert protein staining reagent was used to confirm equal protein loading on the membranes (Figure 2A; *t*_(3__)_ = 0.3738, *p* = 0.73). Next, samples were pre-treated with phosphatase to remove phosphorylation and then probed with Tau1. We confirmed that the overall level of tau was significantly higher in the HP (2.4-fold) when compared to the WM using Tau1 (Figure 2B; *t*_(3)_ = 4.894, *p* = 0.0163), which is remarkably similar to sELISA results above. The dephosphorylated samples show an enrichment of tau in the HP sample compared to the WM sample in all bands with the exception of a slightly lower level in the lowest band. Next, we used MAP2 and PSD-95 as markers known to be enriched in the somatodendritic compartment (Figure 2C). MAP2 and PSD-95 were significantly increased in the HP compared to the WM by 19.6-fold and 2.3-fold, respectively (MAP2: *t*_(3)_ = 7.664, *p* = 0.0046; PSD-95: *t*_(3)_ = 12.01, *p* = 0.0012). We also probed samples for βIII-tubulin, a neuron-specific tubulin isoform that is present in both somatodendritic and axonal compartments and is a major binding substrate for tau. Interestingly, βIII-tubulin signal was significantly increased by 1.3-fold in WM compared to the HP (Figure 2D, *t*_(3)_ = 10.4, *p* = 0.0019), demonstrating that despite the presence of more neuronal microtubules tau levels are not enriched in the WM samples. The enrichment of axonal proteins in the WM samples compared to the HP was confirmed using a myelin marker (MBP) and an axon-specific neurofilament marker (SMI-312), both of which were significantly increased by 13.8-fold and 1.6-fold, respectively (Figure 2E; MBP: *t*_(3)_ = 5.229, *p* = 0.0136; SMI-312: *t*_(3)_ = 9.918, *p* = 0.0022). Collectively, these data suggest that the physiological level of tau ranges between ∼3 and 7 μM across both white and gray matter compartments.

### Tau Distribution in the Adult Rat Hippocampus

**FIGURE 3:**
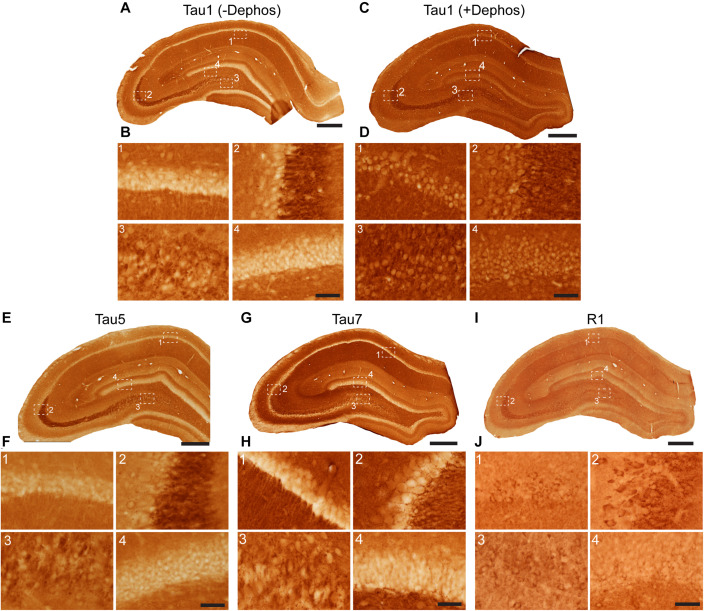
Tau antibodies reveal that tau is normally present in somatodendritic and axonal compartments of neurons in the hippocampus of adult rats. **(A–D)** Tau1 immunostained rat hippocampal brain sections that were either untreated **(A,B)** or treated with alkaline phosphatase to dephosphorylate proteins **(C,D)**. Images from the CA1 (1), CA3 (2), CA3/hilus (3), and dentate gyrus (4) show that without dephosphorylation Tau1 staining is largely restricted to diffuse parenchymal staining reflecting primarily axonal labeling and does not label somatodendritic tau in neurons **(B1–4)**. Tau1 immunostaining is clearly enhanced with dephosphorylation **(C)** and labels robust levels of tau in the somatodendritic compartment of neurons in addition to the parenchymal/axonal staining **(D1–4)**. The intensity of somatic tau appears similar to surrounding parenchyma indicating significant tau levels exist in the somata of neurons. **(E)** Low magnification image of Tau5 immunostaining in the rat hippocampus. **(F)** Images from the CA1 (1), CA3 (2), hilus (3), and dentate gyrus (4) reveal Tau5 stains the parenchymal/axonal tau well and is moderately effective at labeling somatodendritic tau. Somatodendritic labeling with Tau5 was clearest in the CA3 and hilar neurons, although weak diffuse staining was observed in the other regions. **(G)** Low magnification image of Tau7 immunostained rat hippocampal brain sections. **(H)** Images from the CA1 (1), CA3 (2), hilus (3), and dentate gyrus (4) reveal that Tau7 robustly labels parenchymal/axonal tau but shows no signs of labeling tau in the somatodendritic compartment of neurons in rats **(H1–4)**. **(I)** Low magnification image of R1 immunostained rat hippocampal brain sections. **(J)** Images from the CA1 (1), CA3 (2), hilus (3), and dentate gyrus (4) reveal that R1, a polyclonal antibody with epitopes throughout the protein, robustly labels tau in parenchymal/axonal and the somatodendritic compartments of neurons in rats **(J1–4)**. Similar to Tau1, the intensity of somatic tau is similar to surrounding parenchyma and unmyelinated axons of the stratum lucidum mossy fibers in the CA3 region **(J2)** indicating significant tau levels exist in the somata of neurons. Scale bars in panels (**A**,**C**,**E**,**G**,**I)** are 500 μm and in panels (**B4**,**D4**,**F4**,**H4**,**J4)** are 50 μm.

Tau antibodies produced different patterns of staining in the rat HP depending upon the antibody used. Tau1 readily labels parenchymal tau with notable absence of somatodendritic labeling when tissue is not dephosphorylated prior to staining (Figures 3A,B). In contrast, dephosphorylation of the tissue leads to an overall increase in staining across all compartments and a robust labeling of somatodendritic tau (Figures 3C,D). In fact, some of the somatodendritic signals are qualitatively similar to axonal and/or parenchymal staining (e.g., Figure 3D2). This suggests these compartments normally contain similar levels of tau and that axonal tau is not readily phosphorylated at the Tau1 site under physiological conditions, while the tau proteins located in the somatodendritic compartment are phosphorylated at this site. Tau5 labels parenchymal tau and shows a varying degree of somatodendritic tau labeling in different hippocampal cell populations (Figures 3E,F). Notably, Tau7 is exceptional at labeling parenchymal/axonal tau, but shows no reactivity with somatodendritic tau, indicating that its extreme C-terminal epitope is readily available in axons, but not the somatodendritic compartments of neurons (Figures 3G,H). As noted above, we have confirmed that this pattern of reactivity is not due to phosphorylation (Supplementary Figure 3C). Consistent with the polyclonal R1 antibody containing antibodies with multiple epitopes throughout tau, R1 appeared to label all compartments, including axons, dendrites and somata of hippocampal neurons (Figures 3I,J). These findings reveal that tau is normally present in the soma, dendrites and axon of neurons and that labeling tau in each compartment is dependent upon the antibody used (i.e., different epitopes).

**FIGURE 4:**
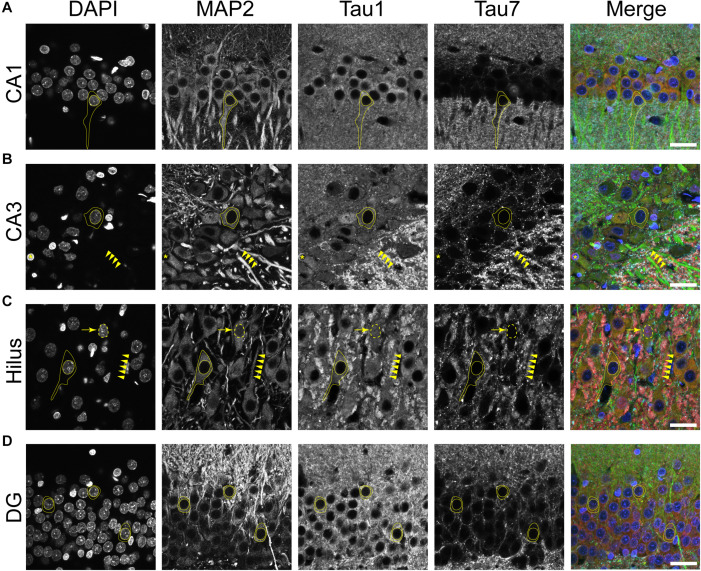
Tau is normally located in cytoplasm, nucleus, dendrites and axons of neurons in the adult rat hippocampus. **(A–D)** To reveal subcellular localization of tau within normal neurons, tissue sections were dephosphorylated by phosphatase treatment and stained with multi-label immunofluorescence for DAPI (nuclear counterstain, blue), MAP2 (somatodendritic marker, green), Tau1 (mid-tau antibody, red), and Tau7 (C-terminal antibody, cyan). Illustrative examples of subcellular localization described below are indicated by yellow shapes/outlines. **(A)** In the CA1 pyramidal cell layer, Tau1 reveals clear presence of tau proteins in the somatodendritic compartment, while the Tau7 antibody does not effectively label somatodendritic tau (outline of a pyramidal neuron that is Tau1+/Tau7-). Notably, the level of Tau1 reactivity with somatodendritic tau is similar to parenchymal levels indicating that the level of tau present in these compartments is similar. **(B)** Staining with Tau1 in the CA3 pyramidal cell layer also reveals clear somatodendritic tau, while Tau7 labels parenchymal tau and does not label somatodendritic tau (outline of neuronal cytoplasm, arrowheads outline a Tau1+/Tau7- apical dendrite). Tau1 clearly labels tau within perineuronal glial cells (yellow asterisks; similar cells noted in other hippocampal regions as well). A notable distinction of tau immunostaining is relatively intense labeling of the cross-sectioned unmyelinated stratum lucidum mossy fibers in the CA3 region, which is typically observed with tau antibodies. **(C)** Similarly, pyramidal neurons in the hilus show clear somatodendritic localization of tau with Tau1 staining and only parenchymal labeling with Tau7 (outline of neuronal cytoplasm and arrowheads outline a apical dendrite). There is clear labeling of tau in the nuclei of some neurons with Tau1 antibody, not Tau7 antibody (arrow and dashed outlined show Tau1+ nucleus; also seen in other regions). **(D)** Dentate granule cells showed clear somatodendritic labeling with Tau1, but Tau7 only stained parenchymal tau (outline of neuronal cytoplasm). Scale bars in merged images are 50 μm.

Multi-label immunofluorescence staining for MAP2 (somatodendritic marker), Tau1 (an antibody that labels dephosphorylated somatodendritic tau), Tau7 (an antibody that does not label somatodendritic tau), and DAPI counterstain (nuclear marker) was used to further evaluate the compartments in which tau is normally distributed in the rat HP. In all hippocampal areas assessed, the CA1-4 pyramidal neurons and dentate granule cells, there was clear evidence of tau in the MAP2+ somatodendritic compartment of neurons as indicated by Tau1 reactivity (tissue was dephosphorylated; Figures 4A–D). In contrast, the extreme C-terminal tau antibody, Tau7, did not effectively label the somatodendritic tau in neurons. A subpopulation of neurons were found that displayed Tau1+ nuclei in the pyramidal neurons (a representative is shown in Figure 4C). No evidence of nuclear staining was present with Tau7. Thus, the differential pattern observed between Tau1 and Tau7 antibodies is a robust illustration of the fact that some antibodies reveal tau in all cellular compartments and some reveal tau in a selective compartment-specific fashion.

**FIGURE 5:**
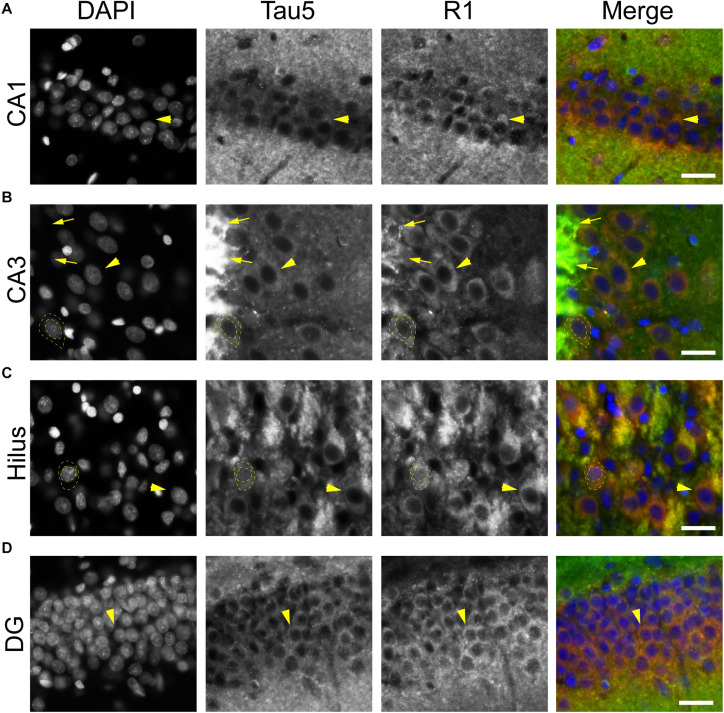
Multiple tau antibodies colocalize within somatodendritic, nuclear and axons of rat hippocampal neurons. **(A–D)** To determine whether different tau antibodies show colocalization with somatodendritic tau in normal neurons, tissue sections were stained with multi-label immunofluorescence for DAPI (nuclear counterstain, blue), Tau5 (mid-tau antibody, green), and R1 (polyclonal antibody, red). Imaging in the CA1 **(A)**, CA3 **(B)**, hilus **(C),** and dentate gyrus **(D)** shows that Tau5 and R1 effectively label somatodendritic tau (arrowheads) and parenchymal tau throughout all rat hippocampal regions. In most cases, the somatodendritic levels of tau are similar to the surrounding parenchyma, with the exception of high signal in the cross-sectioned mossy fibers axons (arrows) in the stratum lucidum of the hippocampus. Note the presence of tau-positive nuclei in a subset of neurons (dashed outlined cell). Scale bars in merged images are 50 μm.

To further validate the distribution of tau in rat hippocampal neurons we performed multi-label immunofluorescence with other tau antibodies. Here, tissue sections were co-labeled with Tau5 (mid-tau antibody) and R1 (rabbit polyclonal antibody; Figure 5). Again, both Tau5 and R1 revealed the clear presence of tau protein within the somatodendritic and axonal compartments of neurons in CA1, CA3, hilus and dentate layers of the HP (Figures 5A–D). Tau5 and R1 labeled a subset of neuronal nuclei in the rat HP (a representative is shown in Figure 5C). As noted above with IHC, Tau5 was moderately effective at labeling somatodendritic tau, and labeled axonal tau quite well (e.g., in the stratum lucidum mossy fibers near CA3, Figure 5B). Similar results were noted in the cerebral cortex with the above antibodies as well (Supplementary Figures 4, 5). Collectively, the multi-label immunofluorescence results presented here qualitatively show that tau is indeed present in the somatodendritic, axonal and nuclear compartment of neurons as indicated by several tau antibodies with different epitopes within the protein, however, some epitopes do not label somatodendritic and nuclear tau under normal conditions (e.g., Tau7 and Tau1 without dephosphorylation).

**FIGURE 6:**
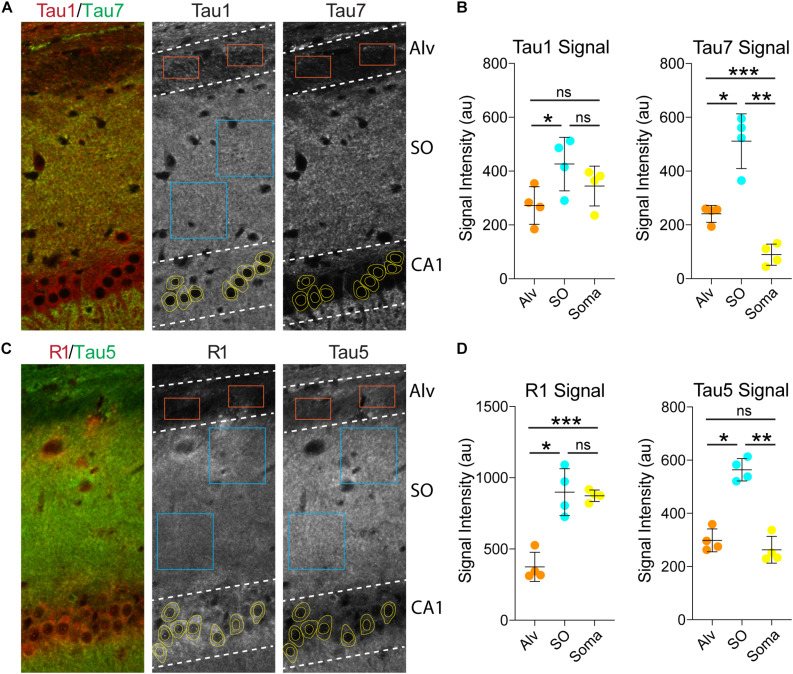
Somatodendritic tau levels are similar or higher than axonal/dendritic regions in the hippocampus. **(A)** Multi-label immunofluorescence images of a rat hippocampus stained with Tau1 (red) and Tau7 (green) antibodies. The grayscale images show representative samplings areas/cells in the alveus (Alv), stratum oriens (SO), and CA1 pyramidal cell layer (CA1). **(B)** Intensity measurement data of Tau1 and Tau7 signals in the Alv, SO and soma. Tau1 signal intensity was significantly greater in the SO compared to the Alv (*p = 0.0165; ns - not significant), but the soma signal was similar to the SO and Alv (ns). Tau7 displayed a markedly different profile of tau labeling. The SO displayed significantly greater signal than the Alv or Soma (*p = 0.0104 and **p = 0.0049), while the soma contained very low signal that was significantly less than the Alv and SO (***p = 0.0023 and **p = 0.0049). **(C)** Multi-label immunofluorescence images of a rat hippocampus stained with R1 (red) and Tau5 (green) antibodies. **(D)** Intensity measurement data of R1 and Tau5 signals in the Alv, SO and soma. R1 shows similar signal intensity in the SO and soma (ns), both of which are significantly greater than in the Alv (***p = 0.0056 and *p = 0.0048). Tau5 displayed yet another distinct profile of tau labeling with the Alv and soma containing similar levels of signal (ns) and the SO containing significantly greater signal than the Alv or Soma (*p = 0.0008 and **p = 0.0002). All comparisons were made using a repeated measures one-way ANOVA with Tukey’s *post hoc* test for multiple comparisons and all graphs represent mean ± SD (*n* = 4/group).

Next, we used the Tau1 and Tau7 (Figures 6A,B) or R1 and Tau5 (Figures 6C,D) immunofluorescence signal to measure the relative intensity of tau labeling in the alveus (contains CA1 pyramidal neuron projection axons), the stratum oriens (contains CA1 pyramidal neuron dendrites and multiple sources of axons), and the CA1 pyramidal neuron somata. Measurements of Tau1 intensity levels showed that the SO region has a relatively small significant elevation of tau compared to the Alv, while the relative Tau1 signal in the soma is similar to the Alv and SO regions (Figures 6A,B; *F*_(1.946, 5.838)_ = 17.45, *p* = 0.0035). All three regions displayed significantly different relative levels of Tau7 staining. The level of Tau7 signal was highest in the SO, followed by the Alv and the lowest levels of tau were observed in the somatodendritic compartment of CA1 neurons (Figures 6A,B; *F*_(1.086, 3.257)_ = 80.75, *p* = 0.0020). The relative intensity of R1 was highest in the SO region and CA1 somata when compared to the Alv layer (Figures 6C,D; *F*_(1.660, 4.981)_ = 46.17, *p* = 0.0007). Finally, Tau5 displayed yet another profile of tau labeling with the Alv and somata containing similar relative levels of signal and the SO containing significantly greater signal than the Alv or Soma (Figures 6C,D; *F*_(1.619, 4.858)_ = 369.7, *p* < 0.0001). Collectively, these semi-quantitative analyses of relative tau levels indicate that somatodendritic tau is similar to or higher than tau levels in non-cellular layers (i.e., containing axons and/or axons and dendrites) with many antibodies (e.g., Tau1, Tau5, R1) and almost undetectable with others (e.g., Tau7). These findings also highlight the diversity in apparent effectiveness of tau antibodies with various epitopes to label the pools of tau in different compartments within neurons.

### Tau Immunostaining Labels Mature Oligodendrocytes in the Rat Brain

**FIGURE 7:**
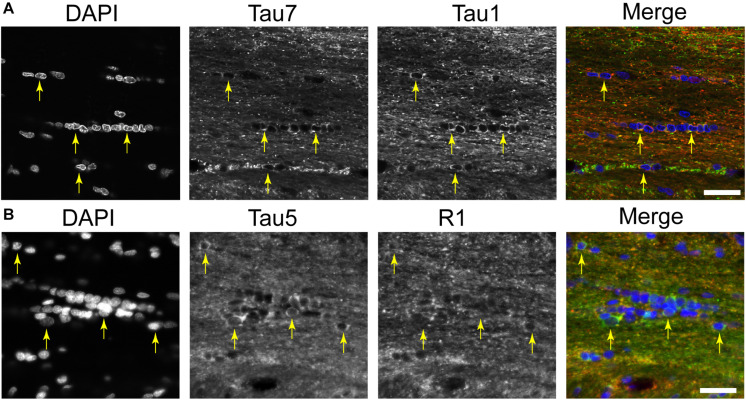
Multiple tau antibodies reveal interfascicular glial cells in adult rat brains. **(A)** Multi-label immunofluorescence staining with DAPI (blue), Tau7 (green), and Tau1 (red) show extensive colocalization with both tau antibodies in interfascicular glial cells (images taken from the DAPI-MAP2-Tau1-Tau7 stained series of tissue). **(B)** Multi-label immunofluorescence staining with DAPI (blue), Tau5 (green), and R1 (red) show some colocalization with both tau antibodies, but R1 does not strongly label interfascicular glial cells (images taken from the DAPI-Tau5-R1 stained series of tissue). Yellow arrows indicate tau positive glial cells. Scale bars in merged images are 50 μm.

A distinct, but often undiscussed, feature in immunostaining with various tau antibodies is the presence of glial cells in both gray matter and WM brain regions (Papasozomenos and Binder, 1987; Migheli et al., 1988; LoPresti et al., 1995; Irving et al., 1996; Tashiro et al., 1997; Takahashi et al., 2000; LoPresti, 2002; Bullmann et al., 2009; Kubo et al., 2019a). Here, we used multi-label immunofluorescence to determine whether different tau antibodies colocalize in glial cells in the rat brain. All of the antibodies tested, including Tau1, Tau7, Tau5, and R1, labeled interfascicular glial cells in the brain (corpus callosum shown as a representative area; Figures 7A,B). Perineuronal glial cells were also noted in hippocampal neuron layers (see Figure 4B).

**FIGURE 8:**
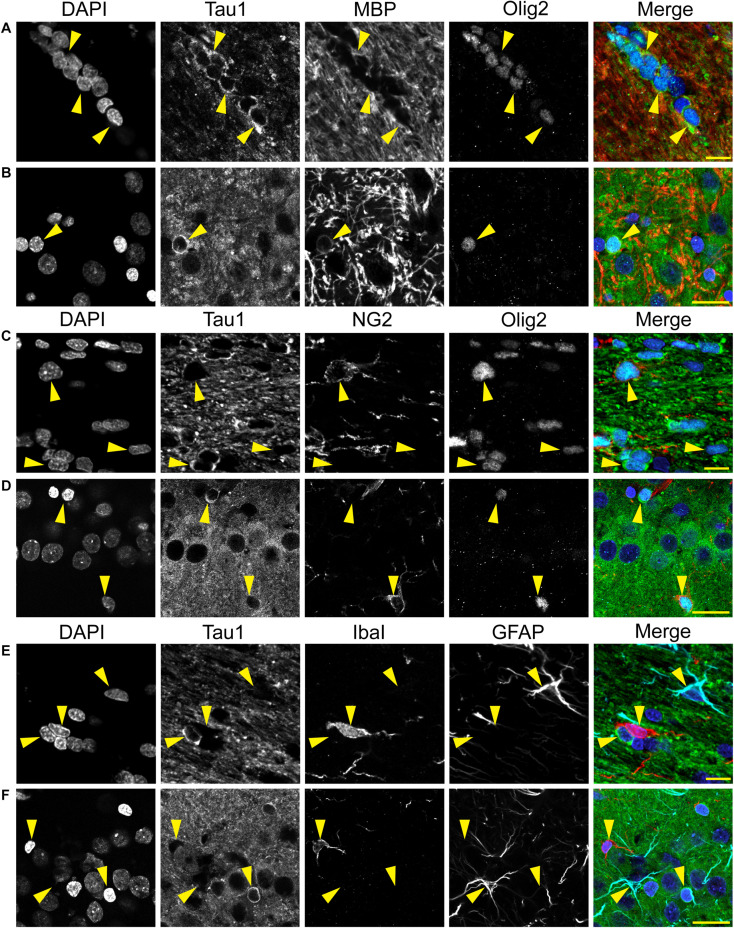
Multi-label immunofluorescence reveals tau positive interfascicular glia are mature oligodendrocytes, not oligodendrocyte precursor cells, astrocytes or microglia, in adult rat brains. **(A,B)** To determine whether interfascicular and perineuronal glial cells containing tau are mature oligodendrocytes, tissue sections were dephosphorylated and stained with multi-label immunofluorescence for DAPI (nuclear counterstain, blue), Tau1 (mid-tau antibody, green), myelin basic protein (MBP, mature oligodendrocyte marker, red), and oligodendrocyte lineage transcription factor 2 (Olig2, an oligodendrocyte marker, cyan). In the corpus callosum **(A)** and hippocampal pyramidal cell layers **(B)**, the Tau1-positive glial cells were also stained with MBP and Olig2 indicating they are mature oligodendrocytes. MBP labeling in tau/Olig2-positive cells was evident, but not all tau/Olig2-positive cells were clearly labeled with MBP suggesting either levels of cytoplasmic MBP were below detection or a subset of unidentified Olig2-positive cells express tau. **(C,D)** To further explore if the tau-positive glial cells were oligodendrocyte precursor cells, tissue sections were stained with multi-label immunofluorescence for DAPI (blue), Tau1 (green), neural/glial antigen 2 (NG2, an oligodendrocyte precursor marker, red), and Olig2 (cyan). In the corpus callosum **(C)** and hippocampal pyramidal cell layers **(D)**, the Olig2-positive cells that were NG2-positive showed little to no tau reactivity indicating that the tau-positive glial cells are not oligodendrocyte precursor cells. Some oligodendrocytes were Olig2-positive but negative for tau and NG2. **(E,F)** To establish whether tau-positive glial cells were astrocytes or microglia, tissue sections were stained with multi-label immunofluorescence for DAPI (blue), Tau1 (green), ionized calcium-binding adapter molecule 1 (IbaI, a microglial marker, red) and glial fibrillary acidic protein (GFAP, an astrocyte marker, cyan). In the corpus callosum **(E)** and hippocampal pyramidal cell layers **(F)**, the tau-positive cells showed little to no co-labeling with GFAP-positive cells or IbaI-positive cells indicating that the tau-positive glial cells are not astrocytes or microglia. Representative cells of each phenotype are indicated with yellow arrows in images. Scale bars in merged images are 10 μm in panels **(A,C,E)** and 20 μm in panels **(B,D,F)**.

We used multi-label immunofluorescence with several glial markers to further identify which populations express tau in the adult rat CNS. First, we stained dephosphorylated tissues with Tau1 (as a representative tau marker), Olig2 (an oligodendrocyte-specific marker), and MBP (a mature oligodendrocyte marker) to establish whether the observed glial cells were mature oligodendrocytes. In white matter (corpus callosum, Figure 8A) and gray matter (HP, Figure 8B), the intensely labeled glial cells were positive for both Olig2 and MBP indicating they are mature oligodendrocytes. Despite relatively low expression of MBP in oligodendrocyte cytoplasm, labeling in tau/Olig2-positive cells was evident but not all tau/Olig2-positive cells were clearly labeled with MBP suggesting either levels of MBP were below detection in the cytoplasm or a subset of unidentified Olig2-positive cells express tau. Next, we performed multi-label immunofluorescence staining with Tau1, Olig2, and NG2 (an oligodendrocyte precursor cell marker) to determine whether tau was expressed in oligodendrocyte precursor cells. Little to no co-localization was observed between Tau1-positive and NG2-positive interfascicular oligodendrocytes in white matter (Figure 8C) or perineuronal oligodendrocytes in the gray matter (Figure 8D) indicating that the tau expressing cells were not oligodendrocyte precursor cells in the normal rat brain. Finally, we used multi-label immunofluorescence with Tau1, GFAP (an astrocyte-specific marker) and IbaI (a microglial marker) to further probe whether the observed tau-positive glial cells were astrocytes or microglia, respectively. Little to no co-localization was observed between Tau1-positive and GFAP-positive cells and there was no indication of tau expression in IbaI-positive microglia in the white matter (Figure 8E) or gray matter (Figure 8F) indicating that the tau expressing cells were not astrocytes or microglia in the normal rat brain. These findings clearly demonstrate that tau is not neuron-specific and that a substantial population of mature oligodendrocytes exists in both white and gray matter that robustly express tau. Furthermore, these data indicate that there is little to no tau expression in oligodendrocyte precursor cells, astrocytes or microglia under physiological conditions.

Our immunohistochemical findings align well with single-nucleus RNA seq data sets such as those from the mouse hippocampus reported by Habib et al. (2016). The online data from this study were searched for *MAPT* transcript levels (Log (TPM) expression) and separated by cell subtypes^2^. The median values were 7.5-6.2 across multiple HP neuronal populations (min = 0, max = 8.8), 8.8 for oligodendrocytes (min = 6.3, max = 9.9), 6.7 for oligodendrocyte precursors (min = 0, max = 8.3), 4.3 for astrocytes (min = 0, max = 7.8), and 0 for microglia (min = 0, max = 5.2) (Habib et al., 2016). Collectively, our results coupled with prior studies at the protein and mRNA level support robust expression of tau in mature oligodendrocytes, not oligodendrocyte precursor cells, astrocytes or microglia.

### Tau Distribution in the Adult Rhesus Macaque Hippocampus

**FIGURE 9:**
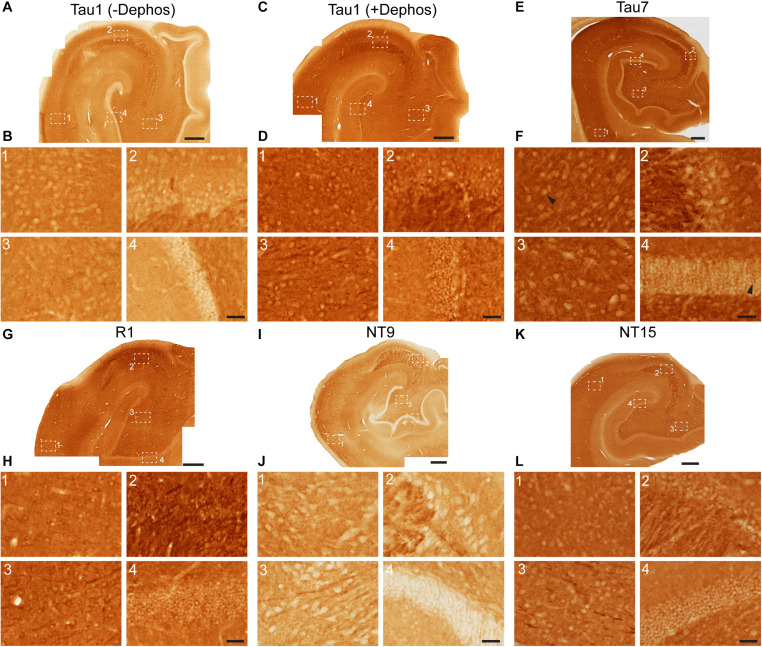
Tau antibodies reveal that tau is normally present in somatodendritic and axonal compartments of neurons in the hippocampus of adult monkeys. **(A–D)** Tau1 immunostained rhesus macaque hippocampal brain sections that were either untreated **(A,B)** or treated with alkaline phosphatase to dephosphorylate proteins **(C,D)**. Images from the CA1 (1) CA3 (2), hilus (3), and dentate gyrus (4) show that without dephosphorylation Tau1 does not readily label somatodendritic tau levels in neurons **(B1–4)**, although weak staining is observed in some areas (e.g., **A1,B1,C1**). However, Tau1 clearly labels robust levels of tau in the somatodendritic compartment of neurons with dephosphorylation **(D1–4)**. Notably, the intensity of somatic tau is similar to surrounding parenchyma and unmyelinated axons of the mossy fibers in the stratum lucidum CA3 layer **(D2)** indicating significant tau levels exist in the somata of neurons. **(E)** Low magnification image of Tau7 immunostained rhesus macaque hippocampal brain section. **(F)** Images from the CA1 (1), CA3 (2), hilus (3), and dentate gyrus (4) reveal that Tau7 robustly labels parenchymal/axonal tau, but does not label somatodendritic tau in the vast majority of neurons **(E1–4)**. Interestingly, some weakly Tau7-positive somata were observed in the CA1 neurons and dentate granule cells in monkeys (black arrowheads). **(G)** Low magnification image of R1 immunostained rhesus macaque hippocampal brain sections. **(H)** Images from the CA1 (1), CA3 (2), hilus (3), and dentate gyrus (4) reveal that R1 robustly labels tau in parenchymal/axonal and the somatodendritic compartments of neurons in monkeys **(H1–4)**. Similar to Tau1, the intensity of somatic tau is similar to surrounding parenchyma and unmyelinated axons of the mossy fibers in the stratum lucidum **(H2)** indicating significant tau levels exist in the somata of neurons. **(I)** Low magnification image of NT9 immunostained rhesus macaque hippocampal brain sections. **(J)** Images from the CA1 (1), CA3 (2), hilus (3), and dentate gyrus (4) reveal that NT9 robustly labels parenchymal/axonal tau, but not somatodendritic tau in hippocampal neurons **(J1–4)**. **(K)** Low magnification image of NT15 immunostained rhesus macaque hippocampal brain sections. **(L)** Images from the CA1 (1), CA3 (2), hilus (3), and dentate gyrus (4) reveal that NT15 labels robust levels of tau in the parenchyma and somatodendritic compartment of neurons in the hippocampus **(L1–4)**. Scale bars in panels **(A**,**C**,**E**,**G**,**I**,**K)** are 500 μm and in panels (**B4**,**D4**,**F4**,**H4**,**J4**,**L4)** are 50 μm.

We used rhesus macaque brain sections to confirm whether the distributions of tau observed in rat hippocampal and WM regions were similar in non-human primates. Tau antibodies produced different patterns of staining in the monkey HP depending upon the antibody used. Tau1 readily labels parenchymal tau with notable absence of somatodendritic labeling when tissue is not dephosphorylated prior to staining (Figures 9A,B). In contrast, dephosphorylation of the tissue leads to an overall increase in staining and a robust labeling of somatodendritic tau (Figures 9C,D). In fact, some of the somatodendritic signals are qualitatively similar to axonal and/or parenchymal staining (e.g., Figure 9D2), suggesting these compartments contain similar levels of tau. Moreover, this indicates that axonal tau is not readily phosphorylated at the Tau1 site under physiological conditions, while the tau proteins normally located in the somatodendritic compartment are phosphorylated at this site. Tau7 is exceptional at labeling parenchymal tau and mostly no reactivity with somatodendritic tau, but very weak somatodendritic staining was observed in a small subset of pyramidal and granule neurons (Figures 9E,F). This suggests that, similar to rat tissue, the extreme C-terminal epitope is readily available in axons but not the somatodendritic compartment of neurons with occasional exceptions to this pattern in monkeys. The R1 antibody labeled all compartments, including axons, dendrites and somata of hippocampal neurons consistent with this reagent containing antibodies with multiple epitopes throughout the tau protein (Figures 9G,H). Somewhat unexpectedly, Tau5 did not react with rhesus macaque tau (Supplementary Figure 2C, see above for protein sequence differences). We used two novel N-terminal antibodies, NT9 (aa 9-44) and NT15 (aa 103-143), to stain tau in monkey tissue sections. The NT9 antibody labeled parenchymal tau quite well but did not readily reveal somatodendritic tau in the monkey HP (Figures 9I,J). The NT15 antibody reacted well with parenchymal tau and labeled somatodendritic localized tau in the monkey hippocampal regions (Figures 9K,L). Similar results were observed in the cortex with the above antibodies (Supplementary Figure 9). Taken together, these findings show that tau is present in the soma, dendrites and axons of neurons under normal conditions in the monkey brain, closely mirroring the results in rat brain.

**FIGURE 10:**
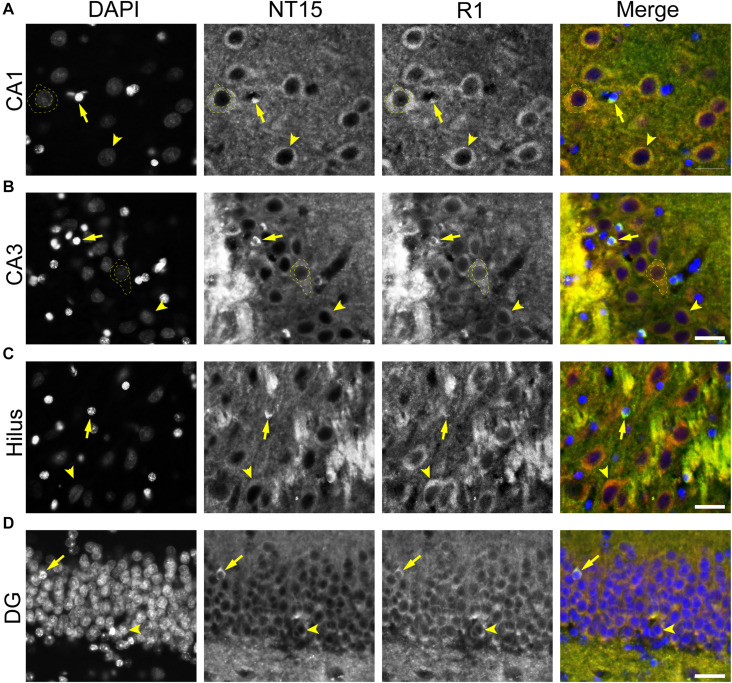
Multiple tau antibodies colocalize with somatodendritic tau in adult rhesus macaque hippocampal neurons. **(A–D)** To determine whether different tau antibodies show colocalization with somatodendritic tau in normal neurons, tissue sections were stained with multi-label immunofluorescence for DAPI (nuclear counterstain, blue), NT15 (mid-tau antibody, green), and R1 (polyclonal antibody, red). Imaging in the CA1 **(A)**, CA3 **(B)**, hilus **(C)**, and dentate gyrus **(D)** shows that NT15 and R1 effectively label somatodendritic tau (yellow arrowhead) and parenchymal tau throughout all monkey hippocampal regions. In most cases, the somatodendritic levels of tau are similar or more intense than the surrounding parenchyma, with the exception of high signal in the cross-sectioned mossy fibers in the stratum lucidum CA3 region of the hippocampus. NT15 and R1 labeled perineuronal oligodendrocytes in all regions of the hippocampus (yellow arrows). R1 showed nuclear tau staining in multiple hippocampal regions, but NT15 did not label nuclei well (yellow dashed outlines). Scale bars in merged images are 50 μm.

To further validate the distribution of tau in monkey hippocampal neurons we performed multi-label immunofluorescence with tau antibodies. Here, tissue sections were co-labeled with NT15 (a primate-specific, N-terminal tau antibody) and R1 (rabbit polyclonal antibody; Figure 10). Both NT15 and R1 revealed the clear presence of tau protein within the somatodendritic and axonal compartments of neurons in CA1, CA3, hilus and dentate layers of the HP (Figures 10A–D). Similar results were noted in the cerebral cortex with these antibodies as well (Supplementary Figures 4, 5). R1 was effective at labeling a subset of neuronal nuclei in the monkey HP, while NT15 did not label nuclear tau well (representative cells are shown in the CA1 and CA3 layers, Figures 10A,B). As noted above NT15 and R1 labeled axonal tau quite well (e.g., in the stratum lucidum mossy fibers, Figure 10B). As in the rat brain, perineuronal oligodendrocytes were robustly labeled with both NT15 and R1 in all hippocampal regions (Figures 10A–D). Collectively, the immunohistochemical and multi-label immunofluorescence results show that tau is indeed present in the somatodendritic, nuclear and axonal compartments of neurons as indicated by several tau antibodies with different epitopes within the protein, however, some epitopes do not label somatodendritic tau under normal conditions (e.g., NT9, Tau7, and Tau1 without dephosphorylation).

### Tau Immunostaining Labels Mature Oligodendrocytes in the Monkey Brain

**FIGURE 11:**
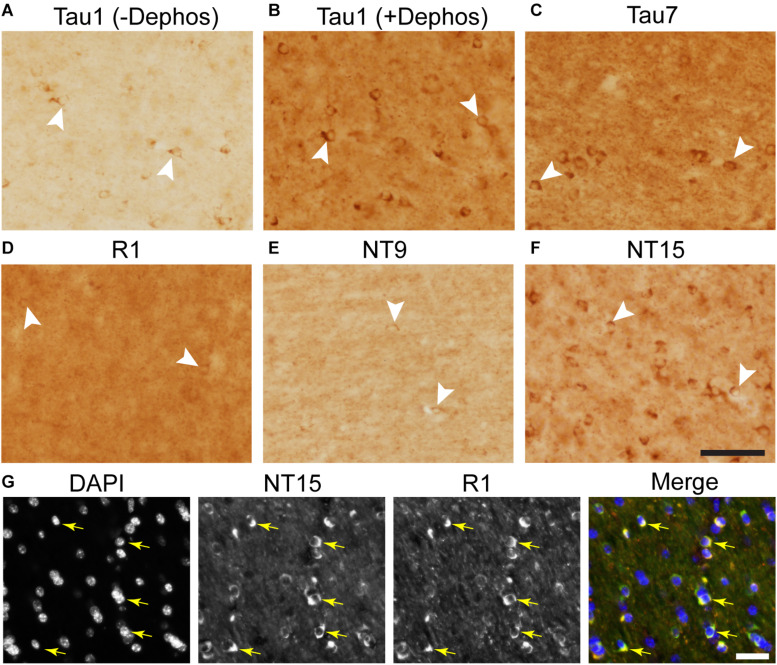
Tau antibodies with epitopes throughout the protein label interfascicular glial cells in adult rhesus macaque brains. **(A,B)** In subcortical white matter of the monkey, Tau1 labels interfascicular glial cells whether tissue sections are untreated **(A)** or treated with phosphatase to dephosphorylate the epitope **(B)**. Tau1 immunoreactivity is clearly increased with phosphatase treatment **(B)**. **(C–E)** Similarly, Tau7 **(C)**, R1 **(D)**, NT9 **(E)**, and NT15 **(F)** all label glial cells in subcortical white matter in monkey. Comparatively, Tau1 (+ dephosphorylation), Tau7 and NT15 are particularly effective among the antibodies tested at labeling interfascicular glial cells in monkeys. White arrowheads indicate positively labeled glial cells. **(G)** Multi-label immunofluorescence staining with DAPI (blue), NT15 (green), and R1 (red) show extensive colocalization with both tau antibodies in interfascicular oligodendrocytes in white matter (images from alveus of the HP). Yellow arrows indicate positively labeled glial cells. Scale bars in panels **(F,G)** (merged) are 50 μm.

**FIGURE 12:**
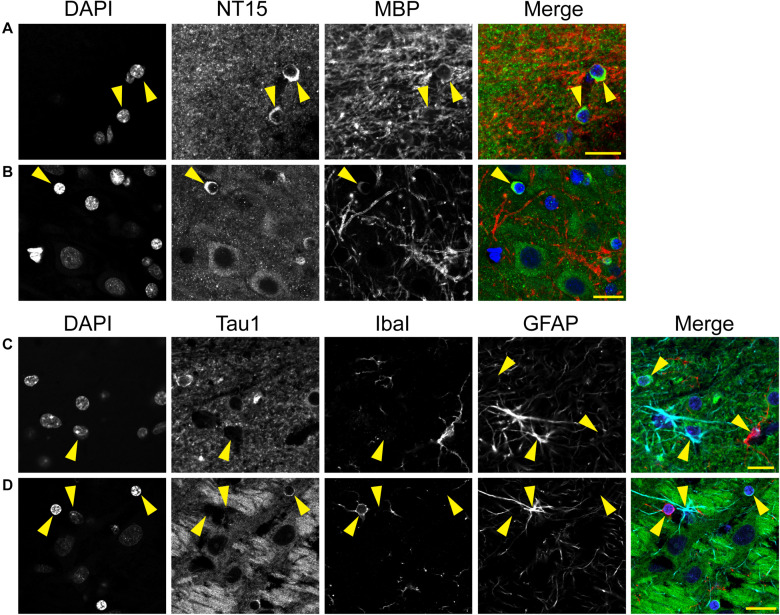
Multi-label immunofluorescence reveals tau positive interfascicular glia are mature oligodendrocytes, not astrocytes or microglia, in adult rhesus macaque brains. **(A,B)** To determine whether interfascicular and perineuronal glial cells containing tau are mature oligodendrocytes, tissue sections were dephosphorylated and stained with multi-label immunofluorescence for DAPI (nuclear counterstain, blue), NT15 (N-terminal tau antibody, green), and myelin basic protein (MBP, mature oligodendrocyte marker, red). In the alveus **(A)** and hippocampal pyramidal cell layers **(B)**, the Tau1-positive glial cells were also stained with MBP indicating they are mature oligodendrocytes. **(C,D)** To establish whether tau-positive glial cells were astrocytes or microglia, tissue sections were stained with multi-label immunofluorescence for DAPI (blue), Tau1 (mid-tau antibody, green), ionized calcium-binding adapter molecule 1 (IbaI, a microglial marker, red), and glial fibrillary acidic protein (GFAP, an astrocyte marker, cyan). In the alveus **(C)** and hippocampal pyramidal cell layers **(D)**, the tau-positive cells showed little to no co-labeling with GFAP-positive cells or IbaI-positive cells indicating that the tau-positive glial cells are not astrocytes or microglia. Representative cells of each phenotype are indicated with yellow arrows in images. Scale bars in merged images are 10 μm in panels **(A,C)** and 20 μm in panel **(B,D)**.

Similar to the rat brain, various tau antibodies reveal the presence of perineuronal oligodendrocytes in hippocampal cell layers (Figures 10A–D) and interfascicular oligodendrocytes in WM (Figure 11). Immunohistochemical staining with Tau1 either with or without dephosphorylation labeled interfascicular glial cells in the alveus of the HP (as well as other WM regions, data not shown), although dephosphorylation robustly improved labeling (Figures 11A,B). In addition, Tau7, R1, NT9, and NT15 labeled interfascicular oligodendrocytes, with Tau7 and NT15 being particularly effective (Figures 11C–F). Finally, multi-label immunofluorescence staining with NT15 and R1 (as representative tau markers) were used to further probe the colocalization of different tau antibodies in glial cells (Figure 11G). In the alveus, both antibodies co-labeled the same cells (Figure 11G). Based upon the detailed glial assessments used in rat tissues, we used multi-label immunofluorescence staining for Tau1 and MBP (a mature oligodendrocyte marker) to establish whether the observed glial cells were mature oligodendrocytes. In white matter (alveus, Figure 12A) and gray matter (HP, Figure 12B), the intensely labeled glial cells were positive for MBP indicating they are mature oligodendrocytes (as in rat brains). It is noteworthy that we tested several Olig2 antibodies (Abcam, ab109186; Abcam, ab42453; IBL, 18953; DSHB, PCRP-OLIG2-1E9-s) and NG2 antibodies (Abcam, ab50009; CST, 43916S; Millipore, MAB2029) in the monkey tissue but none of these reagents worked effectively. While this precluded confirming the lack of tau positivity in oligodendrocyte precursor cells in monkey, we were able to confirm that the tau-positive cells were MBP-positive in monkeys. We used Tau1, GFAP and IbaI to further probe whether the observed tau-positive glial cells were astrocytes or microglia, respectively. Little to no co-localization was observed between Tau1-positive and GFAP-positive cells and there was no evidence of tau expression in IbaI-positive microglia in the white matter (alveus, Figure 12C) or gray matter (HP, Figure 12D) indicating that the tau expressing cells were not astrocytes or microglia in the normal monkey brain. Similar to the rat, these data demonstrate that mature oligodendrocytes in the brain robustly express tau (identified by several tau antibodies), and that astrocytes or microglia do not express detectable tau levels. These findings also confirm that tau is not neuron-specific in the primate brain.

## Discussion

**FIGURE 13:**
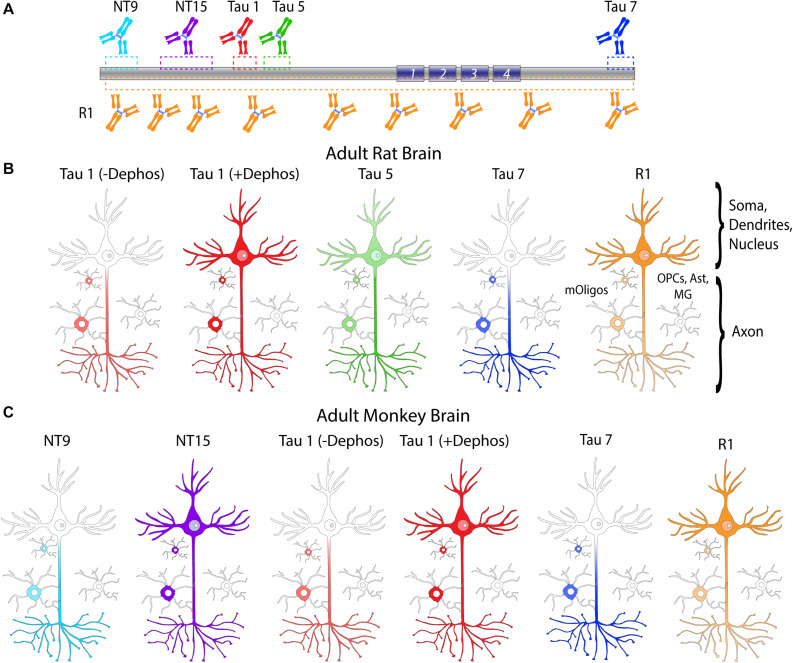
Summary diagram of tau antibodies differentially labeling tau in neurons and oligodendrocytes. **(A)** A diagram of tau protein with the different epitopes indicated for NT9 (IgG1, aa9-44, primate-specific), NT15 (IgG1, aa103-143, primate-specific), Tau1 (IgG2a, aa192-204, requires dephosphorylation), Tau5 (IgG1, aa 218-225, does not react with monkey tau), Tau7 (IgG1, aa430-441), and R1 (rabbit polyclonal, epitopes throughout the tau protein). All tau reagents are specific to tau protein, and with the exception of Tau1, are unaffected by phosphorylation. **(B)** In the adult rat brain, tau antibodies tested effectively label axonal. R1 antibody and Tau1 (with dephosphorylation) effectively label somatodendritic tau and a subset of nuclei, while Tau5 was moderately effective and Tau7 did not readily label tau in these compartments. All tau reagents labeled mature oligodendrocytes (mOligos; MBP+/Olig2+) with Tau1 (with dephosphorylation) and Tau7 being most effective and Tau5 and R1 antibodies less effective. None of the tau antibodies used showed reactivity with NG2+/Olig2+ oligodendrocyte precursor cells (OPCs), astrocytes (Ast; GFAP+) or microglia (MG; IbaI+). **(C)** In the adult monkey brain, all tau antibodies tested effectively label axonal tau. R1, NT15, and Tau1 (with dephosphorylation) effectively label somatodendritic tau, while Tau7 and NT9 did not readily label tau in this compartment. In the monkey, R1 and Tau1 label a subset of neuronal nuclei, as did NT15 albeit weakly. All tau reagents labeled mature oligodendrocytes (mOligos, MBP+) with Tau1 (with dephosphorylation), NT15, and Tau7 being most effective and NT9 and R1 antibodies less effective. None of the tau antibodies used showed reactivity with astrocytes (GFAP+) or microglia (IbaI+). Considering these findings, tau is clearly present in neuronal somata, dendrites, nuclei and axons, as well as mature oligodendrocytes under normal physiological conditions. Thus, tau is not neuron- or axon-specific and exists in substantial quantities in other neuronal compartments and in non-neuronal populations in the brain.

The notion that tau is neuron- and axon-specific remains a pervasive idea in the field despite evidence to the contrary found scattered in the literature dating back to the early studies identifying tau protein localization within cells of the adult CNS (i.e., ∼9 papers from 1987 to 2021 describe correct distribution) (Papasozomenos and Binder, 1987; Migheli et al., 1988; Brady et al., 1995; LoPresti et al., 1995; Tashiro et al., 1997; Gartner et al., 1998; Takahashi et al., 2000; Bullmann et al., 2009; Kubo et al., 2019a). Here, we present a series of tau antibody stains (including two new primate-specific tau reagents) in naïve adult rat and monkey brain tissue, with a focus on the HP as a representative region with relatively well-defined cell body, axon and dendritic regions. In this work, we demonstrated that tau is in-fact normally located within the somata, nuclei, dendrites and axons of neurons, as well as mature oligodendrocytes supporting a wider normal distribution of tau in the adult CNS (Figure 13). As a whole, the data suggest that various epitopes through the tau protein are differentially available for antibody labeling in cellular compartments and whether tau is revealed in these various compartments or cell types is dependent upon the reagents used. Similarities in tau distribution were noted across rat and monkey tissues demonstrating that its presence throughout neurons and in glia is likely conserved from rodents through primates. It is noteworthy that much of the support for axon-specificity of tau is derived from cultured primary neurons where multiple studies highlight the enrichment of tau in the axon of cultured neurons (Mandell and Banker, 1996). However, many axonal populations contain relatively weak tau staining *in vivo* and very few display robust tau signal (see below for additional Discussion) (Kubo et al., 2019a, b). The *ex vivo* nature of primary neuron cultures compared to the intact adult brain is likely a significant limiting factor in using primary neurons to identify physiological distribution of tau in the CNS. Undoubtedly, some of the historical confusion regarding tau distribution in the adult CNS is derived from a “streetlamp” effect where studies using a limited set of reagents miss tau in specific locations because many tau reagents do not fully reveal all pools of the protein in neurons. The relative abundance of tau in different compartments cannot be established using antibodies that do not reveal all pools of tau. Here, we used several antibodies that label tau in all compartments.

Tau is well-known as a flexible, disordered protein with globally folded conformations and relatively extensive post-translational modifications (Friedhoff et al., 2000; Jeganathan et al., 2006; Simic et al., 2016). Each of these characteristics of the tau molecule may directly interfere with antibody reactivity by blocking binding or indirectly by altering tau conformation/structure in ways that obscure the epitope. We cannot rule out that tissue handling or processing artifacts may influence reactivity. Indeed, tau antibody reactivity and the phosphorylation state of tau is impacted by post-mortem interval and other antemortem conditions (Matsuo et al., 1994; Gartner et al., 1998). The rat tissues here were from perfusion-fixed samples and the monkey tissues were saline perfused and immersion fixed samples. The robust labeling and high degree of similarity in the results obtained between rat and monkey tissues suggest the antibodies used here are largely unaffected by these methodological differences. The only notable difference was weak Tau7 labeling of somatodendritic tau in a subset of pyramidal and granule cells in monkey (as a whole the majority of HP neurons were not stained with Tau7), which could be the result of differential fixation methods (i.e., perfusion in rat, immersion in monkey). We confirmed here that all of the tau reagents used are tau-specific and that R1, Tau7, NT9, and NT15 do not appear to be affected by phosphorylation, and Tau5 was already established as a phosphorylation-independent reagent (Carmel et al., 1996).

An interesting pattern appears when comparing various antibodies. For example, antibodies targeting tau termini such as the extreme N-terminal antibodies Tau12, Tau13 (Combs et al., 2016), NT9 (this study) and others (Kubo et al., 2019a, b) or the extreme C-terminal antibody Tau7 (this study) are highly effective axonal tau-specific reagents that do not effectively label the somatodendritic tau. On the other hand, mid-tau antibodies such as Tau1, Tau5 or NT15 are moderately to highly effective at labeling tau in the somatodendritic and axonal compartments. Accordingly, the polyclonal antibody R1, which has epitopes throughout the protein labeled tau in all compartments. Thus, a possible explanation is that tau is differentially modified or folded in local cellular environments that leads to specific patterns of antibody reactivity. Indeed, Tau1 supports this conclusion regarding phosphorylation and comparisons between Tau13 (epitope aa 13-21) to TNT1 (epitope 7-12; as an exemplar) clearly indicate that conformation-dependent differences can dramatically impact reactivity of tau antibodies with directly adjacent epitopes (Kanaan et al., 2011; Combs et al., 2016). Indeed, several antibodies have established the presence of somatodendritic tau. Thus, the lack of reactivity with Tau7 and NT9 (or with any tau antibody) in the somatodendritic compartment does not indicate tau is not there or in low abundance, but instead likely reflects blocked epitopes likely due to conformational differences between axonal and somatodendritic tau or perhaps post-translational modifications other than phosphorylation.

The findings reported here are similar to prior publications showing that neuronal tau is in fact not restricted to the axons. More interesting is the effect the epitope of a specific antibody has on its ability to label tau within discrete sub-neuronal compartments. We measured the relative levels of tau in the somata of CA1 pyramidal neurons, the dendrites/axons of the stratum oriens and the axons of the alveus using immunofluorescence intensity measurements for reactivity with antibodies that effectively label somatodendritic and axonal tau as well as antibodies that only label axonal tau. These analyses indicate that conclusions on tau distribution are directly impacted by the reagent used. For example, Tau1 (with dephosphorylation) and Tau5 show relatively similar levels between the axons of the alveus and CA1 neuron somata, R1 shows much higher soma levels of tau compared with the alveus and Tau7 labels significantly higher levels in the alveus than somata. The collective interpretation of the results is that levels of tau are most likely similar among these different compartments, though they are held in different phospho-statuses (e.g., at the Tau1 epitope). Our findings and those from prior studies (Kubo et al., 2019a, b) have highlighted the robust tau labeling in unmyelinated axons such as the mossy fibers of the stratum lucidum coming into the CA3 region in the HP. Kubo et al. (2019a; 2019b) used reagents that displayed variable detection across axonal populations and did not appear to readily label somatodendritic tau, which precludes the ability to establish differences between somatodendritic and axonal tau. Using qualitative evaluations, they demonstrated that many axonal populations show weak to no detectable tau reactivity, including the corpus callosum, olfactory nerve, anterior commissure, descending corticospinal tracts in the striatum, stria medullaris, cerebellar peduncles, third cranial nerve, medial lemniscus, pyramidal tract, optic nerve, spinal tract of the trigeminal nerve, white matter region of cerebellum (Kubo et al., 2019a). Strong tau reactivity was noted in a few regions, including the mossy fiber axons, molecular layer of the cerebellum, ventral tegmental area, and the stria terminalis. Whether the unmyelinated nature of these axons or other variables influences staining intensity is yet unresolved. It is noteworthy that these few robustly stained axonal populations appear to be the exceptions, not the rule, for tau staining within different WM pathways in the CNS. With that said, it is clear that a few axonal populations within the CNS demonstrate a high level of tau with all antibodies. Supporting similar levels of axonal and somatodendritic tau, our comparisons of the stratum oriens and cell body tau showed similar levels with Tau1 and R1 antibodies. Qualitatively the cell bodies of neurons appear to “disappear” into the surrounding parenchyma with antibodies that effectively label somatodendritic tau suggesting similar levels of tau are present. Collectively, the data do not support the conclusions that basal levels of somatodendritic tau are negligible nor do they support a general enrichment of tau in most axons, although such conclusions could be reached if specific reagents that do not label all tau pools were used (e.g., Tau7 and NT9).

One caveat of the IF densitometry approach is that it is not quantitative, thus, we measured total detergent-soluble tau by quantitative sandwich ELISAs in the rat HP to represent gray matter-enriched tissue and pooled WM (corpus callosum, fimbria and cerebellar peduncles) to represent axon-enriched tissue. We used highly purified recombinant tau protein standards (Combs et al., 2017) instead of brain-derived sources such as those used previously (Binder et al., 1985; Ksiezak-Reding et al., 1988; Khatoon et al., 1992, 1994). The physiological concentration of tau in the rat HP and WM was 6.6 μM and 3.6 μM, respectively. Furthermore, we used immunoblotting to confirm elevated tau in the HP (2.4-fold) compared to the WM, and that the HP samples are enriched for somatodendritic markers (i.e., MAP2 and PSD-95) while the WM samples are enriched for axonal markers (i.e., MBP and SMI-312) as well as βIII-tubulin. When considered in the context of IF densitometry data that allow some degree of compartment specificity, the quantitative sELISA data appear to confirm that tau is unlikely enriched in axons. However, it must be acknowledged that we cannot assign specific concentrations of tau to different cellular compartments with the ELISA approach employed here.

Our molarity data align well with those of Khatoon and coworkers that used radioimmuno-slot-blot assays to measure tau in post-mortem human brain tissue (Khatoon et al., 1992, 1994). Frontal cortex samples contained ∼1 – 2 μg tau/mg protein (similar levels in cerebellar and temporal cortex homogenates) (Khatoon et al., 1994). Using our calculated values of 120 - 130 mg/ml total soluble protein, the tau concentrations are 2.1 and 1.2 μg tau/mg protein in the HP and WM, respectively. Ksiezak-Reding et al. (1988) used semi-quantitative immunoblotting and found tau was 55 – 47 μg/g of tissue in human frontal lobe gray matter and WM samples (as well as the thalamus) from control cases with post-mortem intervals of 2 - 8 h. Using 41.3 kDa, a representative average molecular weight of all tau isoforms (as was used here), and a brain tissue weight to volume of ∼1 g/ml (as was measured here) suggests tau is ∼1 μM. We also found that tau was ∼2 times higher in gray matter-enriched sample (i.e., HP) compared to WM-enriched samples (combined corpus callosum, fimbria and cerebellar peduncles) when assessing total detergent-soluble protein lysates. In contrast, Binder et al. (1985) used twice-cycled microtubule fractions from the bovine caudate (gray matter) and internal capsule (WM) and found that tau was 0.08 mg/ml in gray matter and 0.19 mg/ml in the WM (measured with competitive ELISAs). Using 41.3 kDa molecular weight yields 1.9 μM tau in gray matter and 4.6 μM in WM. The use of twice-cycled microtubules to enrich tau likely accounts for the somewhat lower molarity. The differences in gray matter versus WM using cycled microtubules may reflect a higher binding affinity of axonal tau for axonal microtubules but this requires further investigation. Neither Khatoon et al. (1994) nor Ksiezak-Reding et al. (1988) found a difference in tau levels between gray and WM. One must also consider that, in the approaches used by us and others, neither sample is purely somatodendritic or axonal precluding definitive neuron- or axon-specific measures. In addition, the approaches used cannot effectively distinguish between intracellular and extracellular tau, although the level of extracellular tau is quite low (∼1 nM, ∼45 ng/ml) under normal conditions (Yamada et al., 2011). Collectively, our quantitative findings and those of others indicate that the historical range of 1 – 4 μM should be revised to ∼3 – 7 μM.

We used hippocampal subregions as representative regions in the rat and monkey brain, but the general findings of neuronal and glial localization of tau was similar in several other brain regions such as the cortex, subcortical regions and brainstem areas (NMK personal communication) as reported by others (Papasozomenos and Binder, 1987; Migheli et al., 1988; Brady et al., 1995; LoPresti et al., 1995; Tashiro et al., 1997; Gartner et al., 1998; Takahashi et al., 2000; Bullmann et al., 2009; Kubo et al., 2019a). The presence of tau in axons is one of the best studied compartmental pools of tau and studies indicate a clear role in regulating axonal functions such as transport (Kanaan et al., 2015b; Kneynsberg et al., 2017), microtubule dynamics (Qiang et al., 2018), and neurite outgrowth (Mandell and Banker, 1996). The nuclear presence of tau also provides interesting insights into the alternative biological functions of tau. The relatively sporadic presence of nuclear tau among the hippocampal cell populations analyzed here under normal physiological conditions is a phenomenon for which the functional implications are currently not well understood. However, studies suggest that nuclear pools of tau are potentially involved in stabilizing chromatin, nucleolar organization and/or protecting DNA and RNA from damage (Sjöberg et al., 2006; Wei et al., 2008; Sultan et al., 2011; Camero et al., 2014; Violet et al., 2014). We did not have the resolution to identify individual synapses in this work. Other studies have suggested the presence of tau in dendritic spines and synapses, which may have a role in tau-mediated dysfunction in disease (Ittner et al., 2010; Mondragon-Rodriguez et al., 2012; Tai et al., 2012; Frandemiche et al., 2014). However, a lack of tau in synapses and dendritic spines was noted under normal conditions using Tau1 labeling in transmission electron microscopy analysis (Papasozomenos and Binder, 1987).

Numerous non-neuronal tau inclusions characterize human tauopathy diseases, including coiled bodies in oligodendrocytes and several variations of astrocytic inclusions, while there is very little evidence of microglia accumulating tau pathology (Ikeda et al., 1998; Arai et al., 2001; Ghoshal et al., 2001; Ferrer et al., 2014). However, the normal expression of tau in glial cells is not well-studied and often disregarded as insignificant, which is not in agreement with transcript- and protein-level analyses. Single-cell and single-nucleus RNA-seq studies from normal mice show a pattern of *MAPT* expression that is highest in mature oligodendrocytes, intermediate in neurons and oligodendrocyte precursor cells, low in astrocytes and even lower in microglia (Habib et al., 2016; Ximerakis et al., 2019). Other studies have shown oligodendrocytes express tau mRNA as well (LoPresti, 2002). The presence of tau protein in oligodendrocytes was consistently revealed by all the antibodies tested here (NT9, NT15, Tau1, Tau5, Tau7, and R1), which aligns quite well with the prior studies reporting clear tau positive oligodendrocytes (Papasozomenos and Binder, 1987; Migheli et al., 1988; LoPresti et al., 1995; Irving et al., 1996; Tashiro et al., 1997; Takahashi et al., 2000; Bullmann et al., 2009; Kubo et al., 2019a). Among the reagents tested, R1 (both rat and monkey), Tau5 (rat only), and NT9 (monkey only) were least effective in detecting oligodendrocytes in the brain. Although glia are significantly smaller than neurons, the intensity of labeling suggest there is a substantial level of tau protein expressed in these cells. We cannot exclude the possibility that oligodendrocytes are accumulating tau secreted by neurons, however, the fact that oligodendrocytes display high levels of tau transcripts (LoPresti, 2002; Habib et al., 2016; Ximerakis et al., 2019) and the interstitial tau levels are relatively low under physiological conditions (Yamada et al., 2011) make this explanation unlikely.

Interestingly, the tau antibodies used here labeled a subset of the oligodendrocytes, which led us to further identify specific glial subtypes. We used MBP to show that the intensely labeled glial cells were mature oligodendrocytes (in rats and monkeys) and the lack of co-localization with NG2 demonstrated that the tau expressing cells were not NG2-positive oligodendrocyte precursor cells. These data suggest tau expression is robustly increased once oligodendrocytes have differentiated into a mature phenotype, however, the underlying functional implications of tau expression in these cells remains undefined. Interestingly, a prior study noted that the level of tau positive oligodendrocytes was significantly increased following administration of glutamate (Irving et al., 1996) raising the possibility that local levels of neurotransmitters and/or neuron activity may modulate tau expression in oligodendrocytes. We also used GFAP or IbaI to determine whether the tau-positive glial cells were astrocytes or microglia, respectively. There was little to no evidence of tau expression in astrocytes or microglia in the normal adult rat and monkey brain. Consistent with our findings, some prior studies noted a lack of clear tau protein expression in astrocytes (Kubo et al., 2019a), while other studies show astrocytic tau expression (Papasozomenos and Binder, 1987). Our results and those from others suggest that tau expression in astrocytes is likely low and/or transient under normal conditions, and may increase under pathological conditions where astrocytic tau inclusions are present. There is very little to no evidence supporting the expression of tau in microglia cells in the CNS (Habib et al., 2016; Ximerakis et al., 2019). However, at least one study showed that microglia may accumulate some forms of pathological tau in disease or disease models (Ghoshal et al., 2001) and a potential relationship between tau pathologies and microglia in human disease processes are suggested (Serrano-Pozo et al., 2011; Kahlson and Colodner, 2015; Španić et al., 2019). The physiological relevance of glial tau expression remains unknown and whether brain region-specific, activity-dependent or yet unidentified variables cause differences in tau expression among glial populations is not established, but the data clearly point to the fact that mature oligodendrocytes in fact express significant levels of tau protein under normal conditions.

The work presented here has important implications for the interpretation of experimental data and formulation of hypotheses related to tau biology and pathobiology. For example, the hypothesis that tau undergoes an abnormal redistribution or mislocalization from the axon to the somatodendritic compartment is often discussed as a key pathological step in tauopathies such as Alzheimer’s disease (Ittner and Götz, 2011; Zempel and Mandelkow, 2014). Support for the abnormal redistribution of tau in disease stems primarily from the idea that tau is normally absent or kept at negligible levels in the soma and dendrites and high levels in axons. The evidence presented here and elsewhere clearly eliminate this supporting tenet. However, the presence of somatodendritic tau does not directly negate the possibility that tau redistribution might occur in human disease and contribute to tau-mediated dysregulation or toxicity. Indeed, there is compelling evidence that the axon initial segment may normally act as a one-way gate for axon-destined tau proteins and that disease processes and/or disease-related tau modifications may interfere with this functionality (Li et al., 2011; van Beuningen et al., 2015; Zempel et al., 2017). However, this does not sufficiently prove tau mislocalization is the driving force behind the accumulation of abnormal tau species in the somatodendritic compartment, where tau does in fact, normally reside at substantive levels. Moreover, the clear presence of abundant tau in mature oligodendrocytes requires that we no longer consider tau a neuron-specific protein and that tau likely plays an important functional role in oligodendrocytes under normal conditions and disease conditions. Thus, there is a need for additional studies that specifically address these important unresolved issues. Finally, the data presented here and in prior studies collectively indicate that caution must be exercised when drawing conclusions on tau distributions (both normal and pathological) and/or in generating hypotheses about tau localization. The use of multiple tau antibodies and/or reagents that are validated to highlight tau in all relevant cellular compartments or cell types is necessary.

## Data Availability Statement

The raw data supporting the conclusions of this article will be made available by the authors, without undue reservation.

## Ethics Statement

The animal study was reviewed and approved by the Michigan State University, Institutional Animal Care and Use Committee (rodents) & Rush University Medical Center and the Biological Research Laboratory at the University of Illinois Chicago Institutional Animal Care and Use Committee (non-human primates).

## Author Contributions

NK and TG performed all aspects of the work including experimentation, data collection and presentation, and wrote the manuscript. Both authors contributed to the article and approved the submitted version.

## Conflict of Interest

The authors declare that the research was conducted in the absence of any commercial or financial relationships that could be construed as a potential conflict of interest.

## Footnotes

1 www.uniprot.org

2 https://singlecell.broadinstitute.org/single_cell/study/SCP1/-single-nucleus-rna-seq-of-cell-diversity-in-the-adult-mouse-hippocampus-snuc-seq#study-summary
