# Supplementary material for: Neuronal and Glial Distribution of Tau Protein in the Adult Rat and Monkey
Source: Front Mol Neurosci. 2021 Apr 27;14:607303. doi: 10.3389/fnmol.2021.607303 (PMC8112591; doi:10.3389/fnmol.2021.607303)
Supplement: Supplementary file 1 [file Data_Sheet_1.docx]

Supplementary Material

# Supplementary Figures


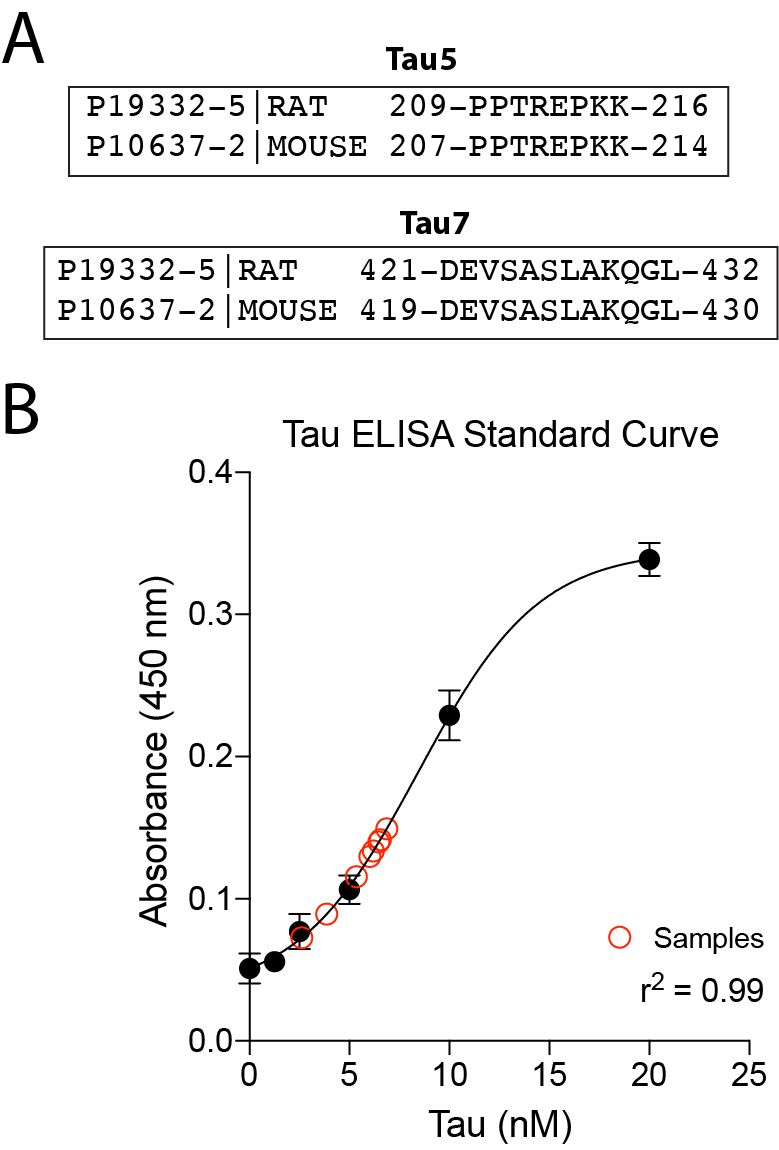


**Supplementary Figure 1.** Quantitative Sandwich ELISA Measurements. **(A)** Protein sequence alignments between rat and mouse tau at the Tau5 and Tau7 antibody epitopes. Mouse and rat tau have 100% homology at these epitopes. **(B)** Standard curve of recombinant mouse tau (0 - 20 nM) in the quantitative sandwich ELISA where Tau7 was used as the capture antibody and biotinylated Tau5 was used as the detection antibody. The standards were analyzed using a nonlinear sigmoidal fit curve (r^2^ = 0.99) and unknown brain samples were interpolated from the curve (open red circles).


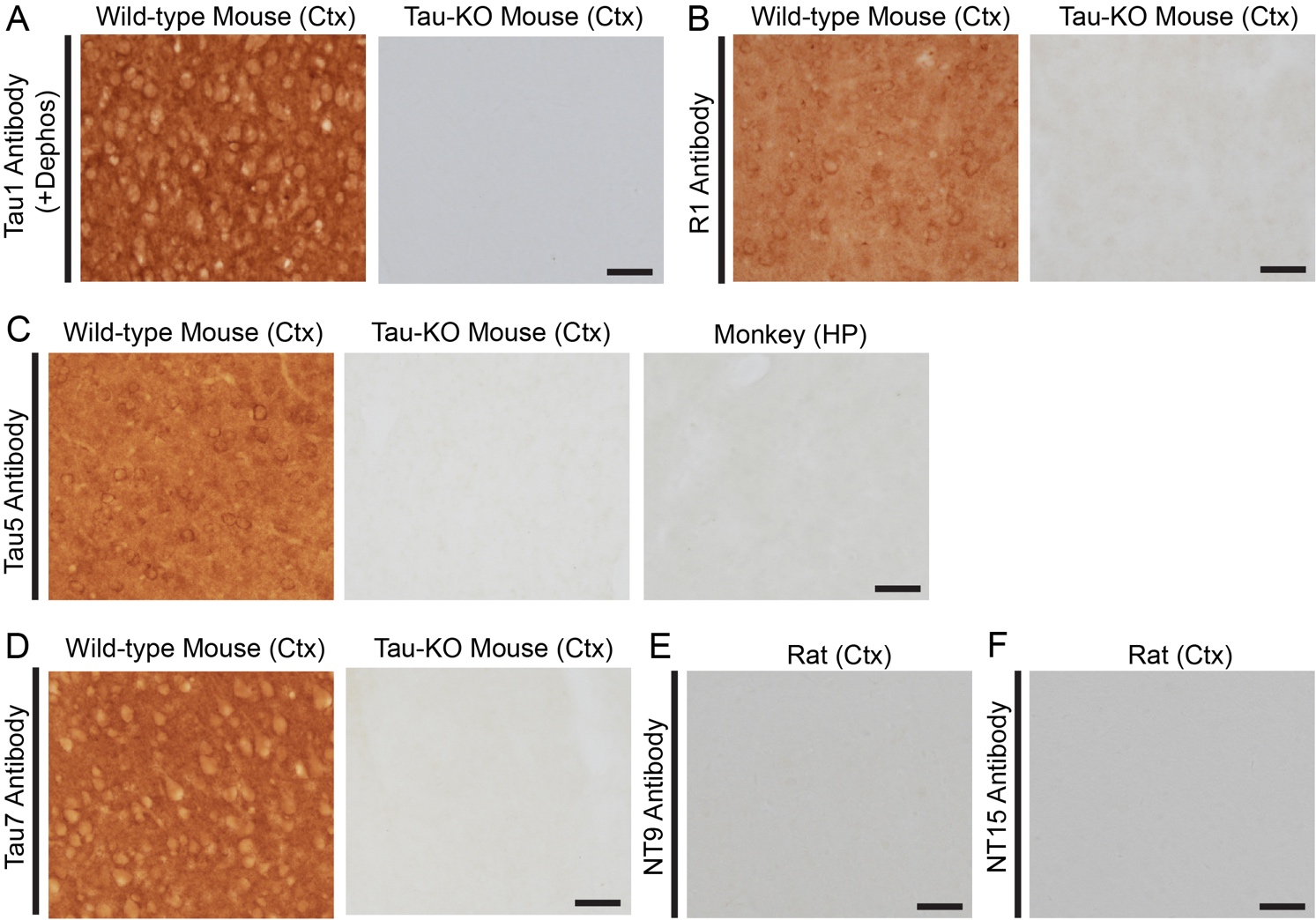


**Supplementary Figure 2.** Tau antibody specificity validation. **(A)** Wild-type (C57/BL6J) and tau knockout mouse (Tau-KO) sections containing the cortex (Ctx) were used in immunohistochemical staining procedures with phosphatase treatment (+Dephos) for Tau1 immunostaining as described in the methods section. Typical tau immunoreactivity was observed in wild-type mice, but no signal was detected in Tau-KO mice indicating that the Tau1 antibody is specific for tau protein. **(B)** The R1 antibody was used to stain wild-type and Tau-KO sections containing the cortex. Typical tau immunoreactivity was observed in wild-type mice, but no signal was detected in Tau-KO mice indicating that the R1 antibody is specific for tau protein. **(C)** The Tau5 antibody was used to stain wild-type and Tau-KO sections containing the cortex. Typical tau immunoreactivity was observed in wild-type mice, but no signal was detected in Tau-KO mice. In addition, hippocampal (HP) monkey sections were stained with Tau5, which does not react with monkey tau. Together these results indicate that the Tau5 antibody is specific for tau protein, but not in monkey. **(D)** The Tau7 antibody was used to stain wild-type and Tau-KO sections containing the cortex. Typical tau immunoreactivity was observed in wild-type mice, but no signal was detected in Tau-KO mice indicating that the Tau7 antibody is specific for tau protein. **(E-F)** The novel NT9 **(E)** and NT15 **(F)** antibodies were used to stain rat sections containing the cortex. No signal was detected in rats, in contrast to the typical tau staining observed in monkey sections (see **Figures 8-11**) indicating that the NT9 and NT15 antibodies are specific for tau protein in primates (i.e. monkey, and humans – see **Supplementary Figure 3**). The results of these immunohistochemical controls also indicate that the staining procedures used do not produce non-specific background signal. Scale bars are 50 μm.

­
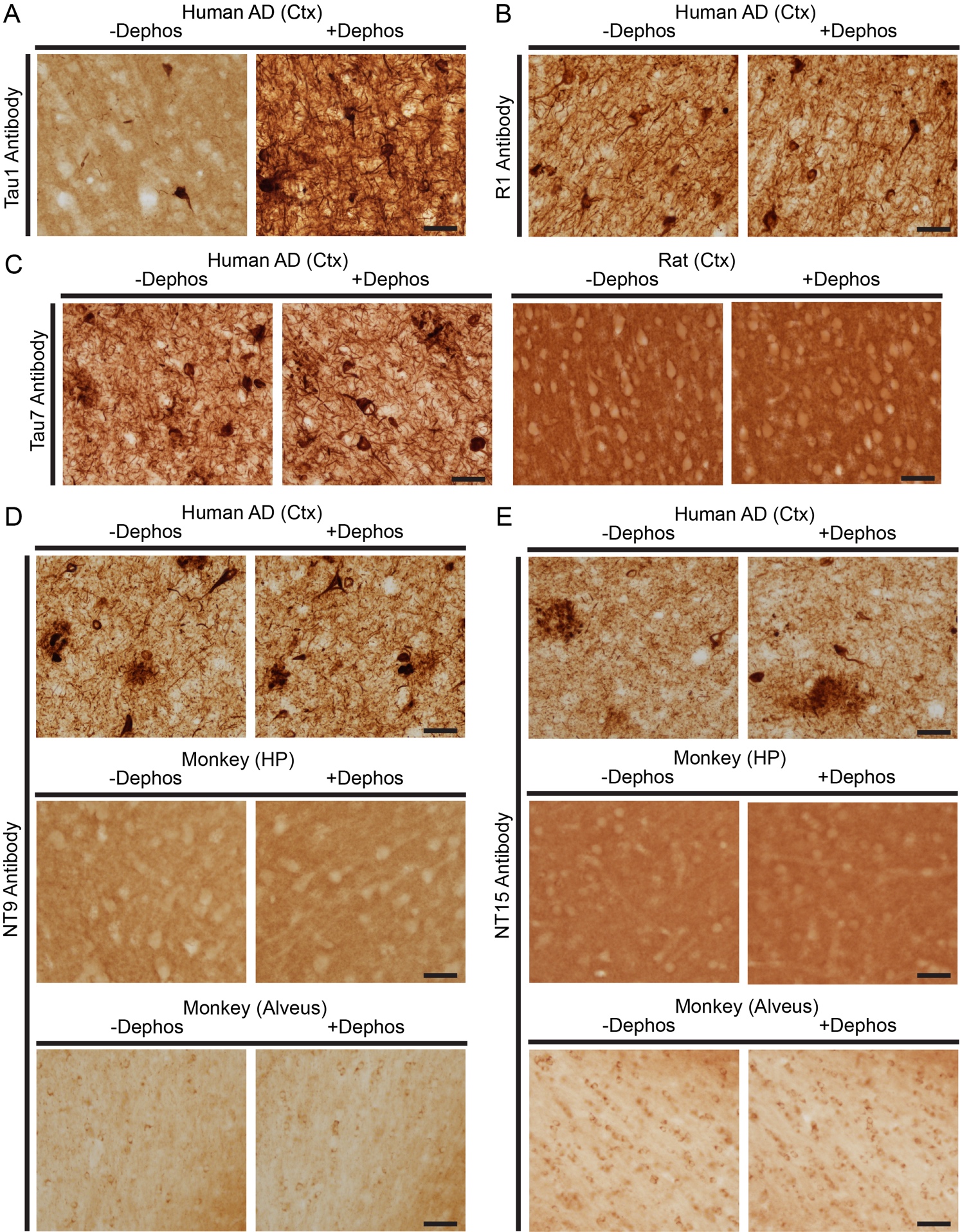


**Supplementary Figure 3.** Determination of whether phosphorylation of tau impacts reactivity with Tau1, Tau7, R1, NT9 and NT15 immunoreactivity. **(A)** The Tau1 antibody is established as an antibody that requires dephosphorylation to reveal the extent of tau pathology present in Alzheimer’s disease (AD) brain and was used here as a positive control for the effectiveness of phosphatase treatment (+Dephos). As expected, Tau1 reactivity is minimal in untreated tissue sections (-Dephos) from an AD case (Braak stage V-VI, inferior temporal gyrus, Ctx). In contrast, robust pathology was revealed when sections from the same case and region were pretreated with phosphatase, confirming the dephosphorylation procedure. **(B-E)** In contrast to Tau1, neither R1 **(B)**, Tau7 **(C)**, NT9 **(D),** nor NT15 **(E)** showed any discernable differences between untreated and phosphatase treated sections in human AD. **(C)** In addition, rat sections containing the cortex (Ctx) were either untreated (-Dephos) or treated with phosphatase (+Dephos) to establish whether phosphorylation status of tau impacted Tau7 staining in normal rat tissue. There was no appreciable difference in Tau7 immuoreactivity between untreated or treated sections indicating Tau7 is not affected by phosphorylation. **(D,E)** For NT9 **(C)** and NT15 **(D)**, monkey sections containing the hippocampus (HP) and alveus were either untreated (-Dephos) or treated with phosphatase (+Dephos) to establish whether phosphorylation status of tau impacted staining in the normal monkey tissue. Neither the CA1 HP region (representative grey matter area) nor the alveus (representative white matter area) showed discernable differences in the intensity or pattern of immunoreactivity. Taken together, these data confirm the phosphorylation dependence of Tau1 and establish that reactivity with the R1, Tau7, NT9 and NT15 antibodies is independent of phosphorylation. Scale bars are 50 μm.


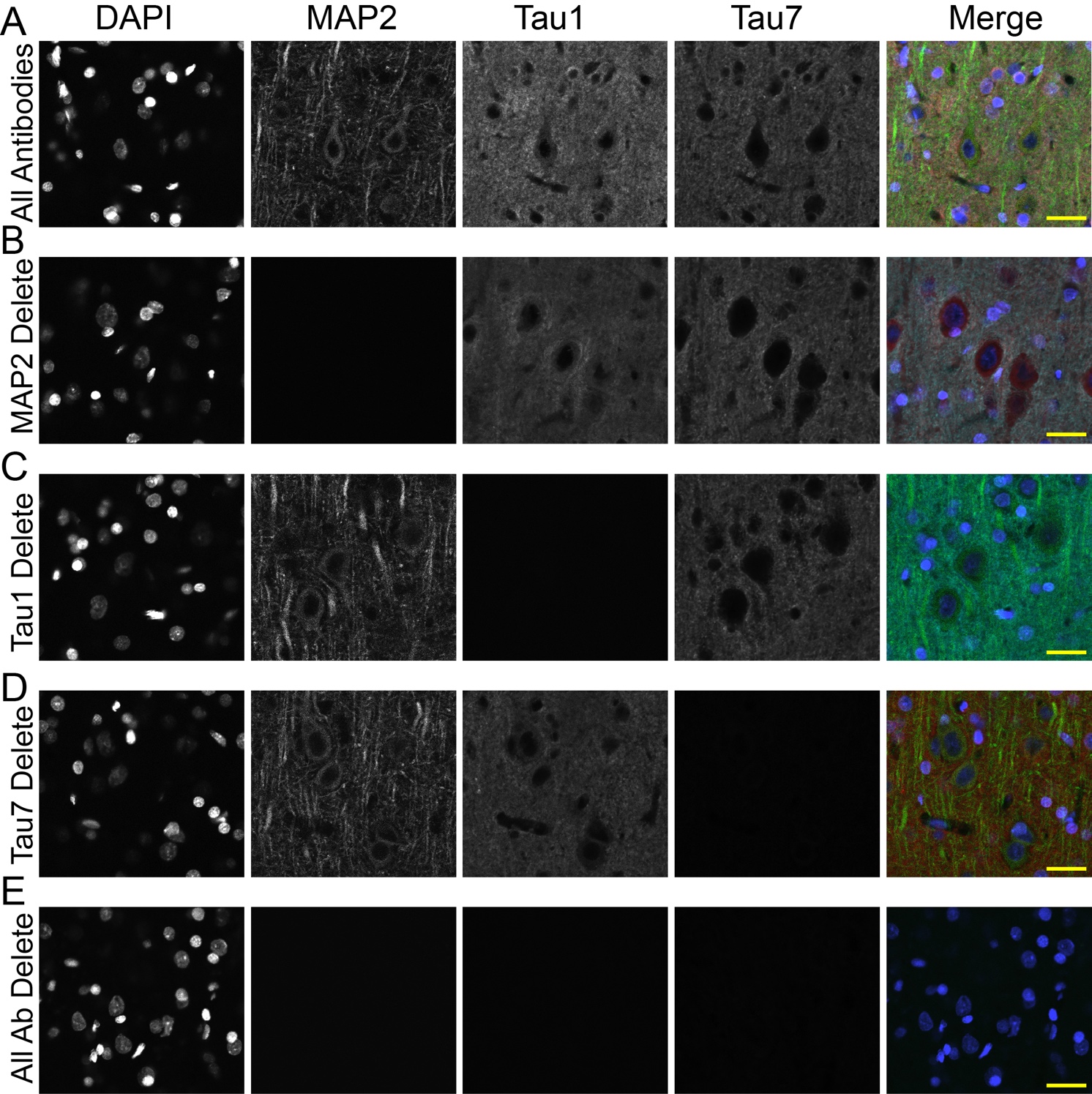


**Supplementary Figure 4.** Primary antibody delete controls for MAP2, Tau1 and Tau7 multi-label immunofluorescence in rat tissue. **(A)** Tissue sections (cortex depicted) were stained with MAP2, Tau1, Tau7 and counterstained with DAPI. **(B-E)** Sections were processed following the same procedures with the exception of omitting either MAP2 **(B)**, Tau1 **(C)**, Tau7 **(D)** or all three tau primary antibodies **(E)**. Note the lack of staining cross-over in individual primary deletes or non-specific signal in the full primary delete demonstrating specific detection of each tau antibody. As in the hippocampus, Tau1 labels somatodendritic tau in neurons, while Tau7 does not. Scale bars are 50 μm.


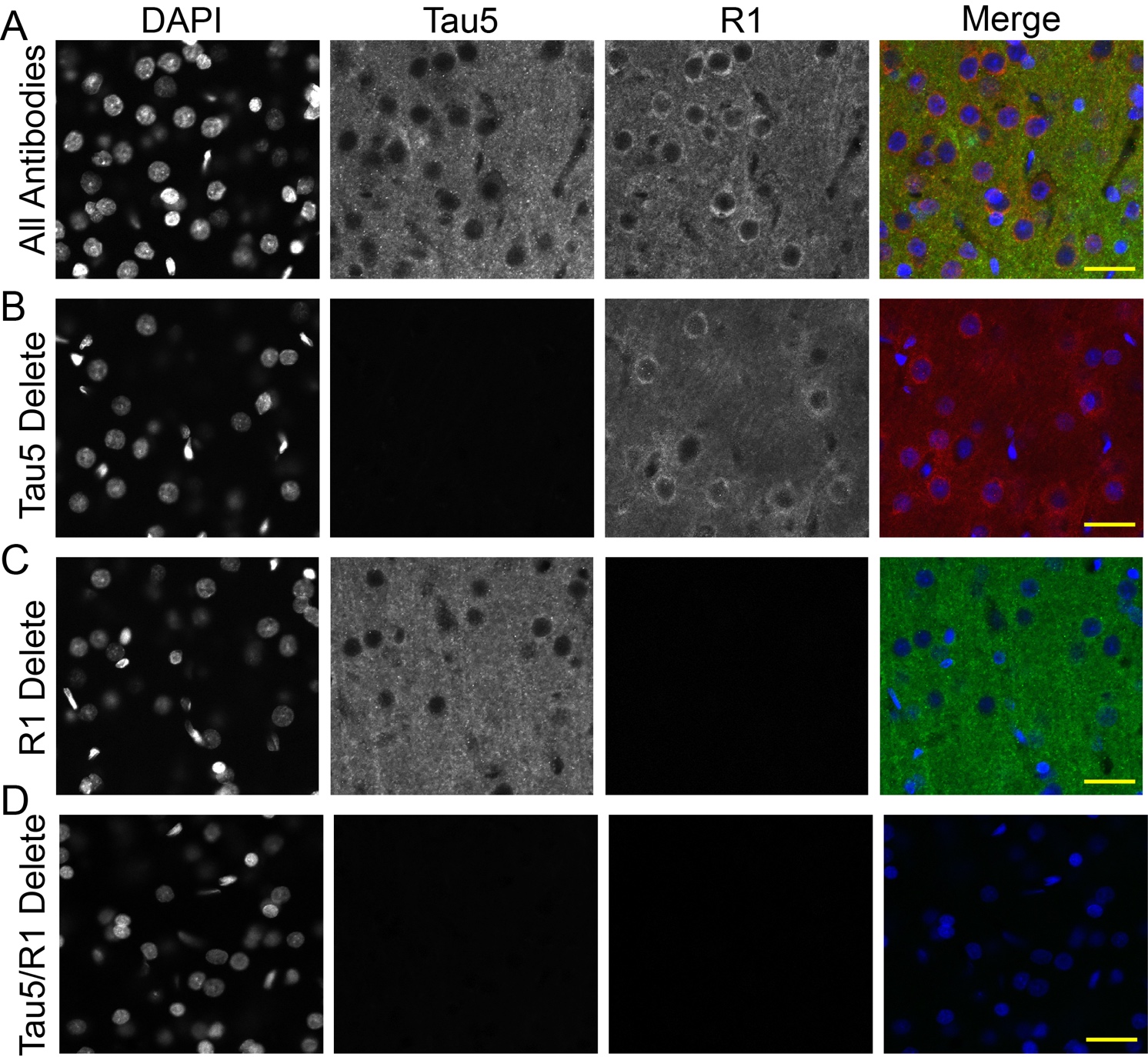


**Supplementary Figure 5.** Primary antibody delete controls for Tau5 and R1 multi-label immunofluorescence in rat tissue. **(A)** Tissue sections (cortex depicted) were stained with Tau5, R1 and counterstained with DAPI. **(B-D)** Sections were processed following the same procedures with the exception of omitting either Tau5 **(B)**, R1 **(C)** or both tau primary antibodies **(D)**. Note the lack of staining cross-over in individual primary deletes or non-specific signal in the full primary delete demonstrating specific detection of each tau antibody. As in the hippocampus, Tau5 and R1 label somatodendritic tau in neurons. Scale bars are 50 μm.


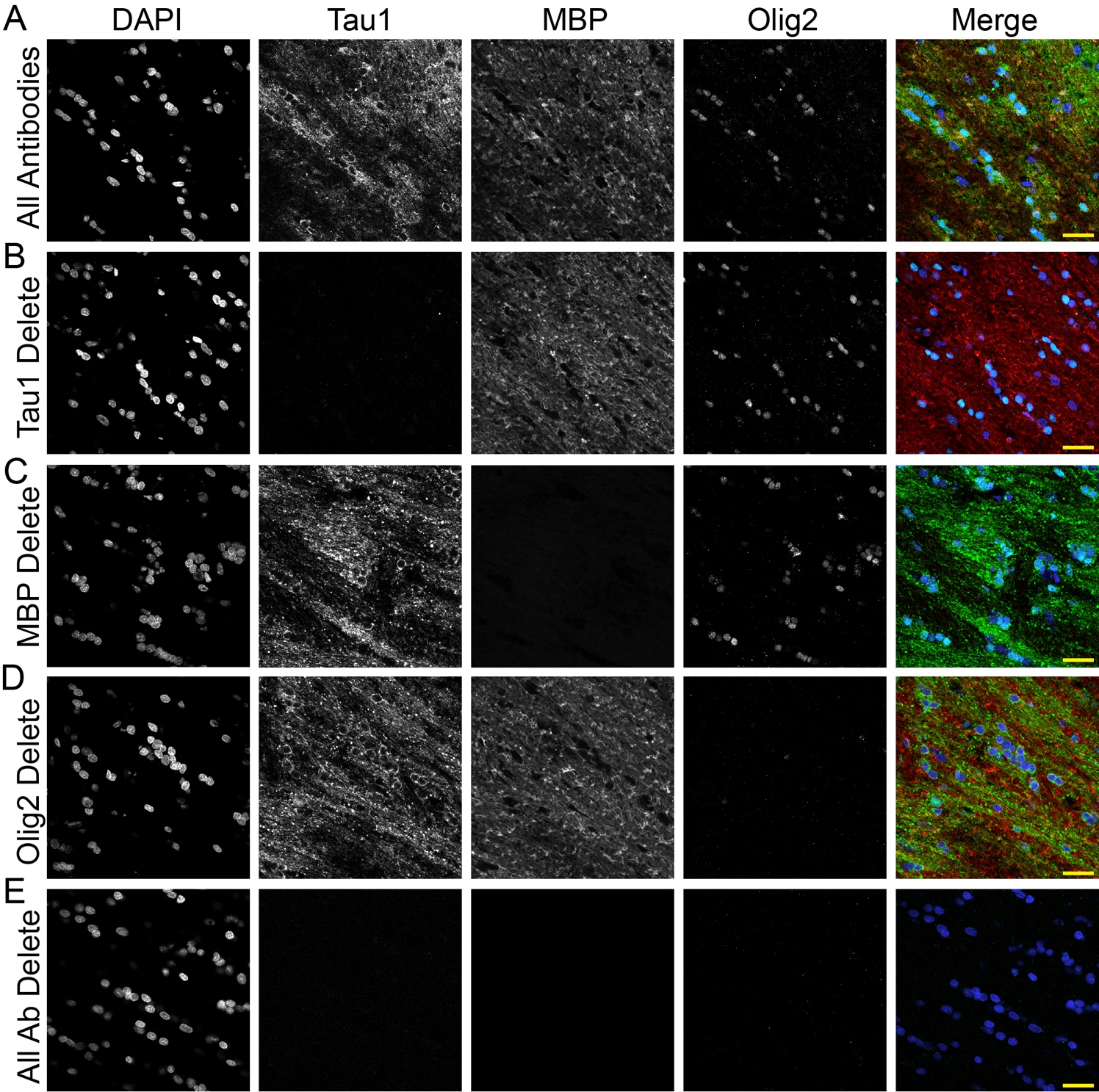


**Supplementary Figure 6.** Primary antibody delete controls for Tau1, MBP and Olig2 multi-label immunofluorescence in rat tissue. **(A)** Tissue sections (corpus callosum depicted) were stained with Tau1, MBP and Olig2 and counterstained with DAPI. **(B-E)** Sections were processed following the same procedures with the exception of omitting either Tau1 (**B**), MBP **(C,** mature oligodendrocyte marker**)**, Olig2 (**D**, oligodendrocyte marker) or all primary antibodies **(E)**. Note the lack of staining cross-over in individual primary deletes or non-specific signal in the full primary delete demonstrating specific detection of each antibody. Scale bars are 25 μm.

**
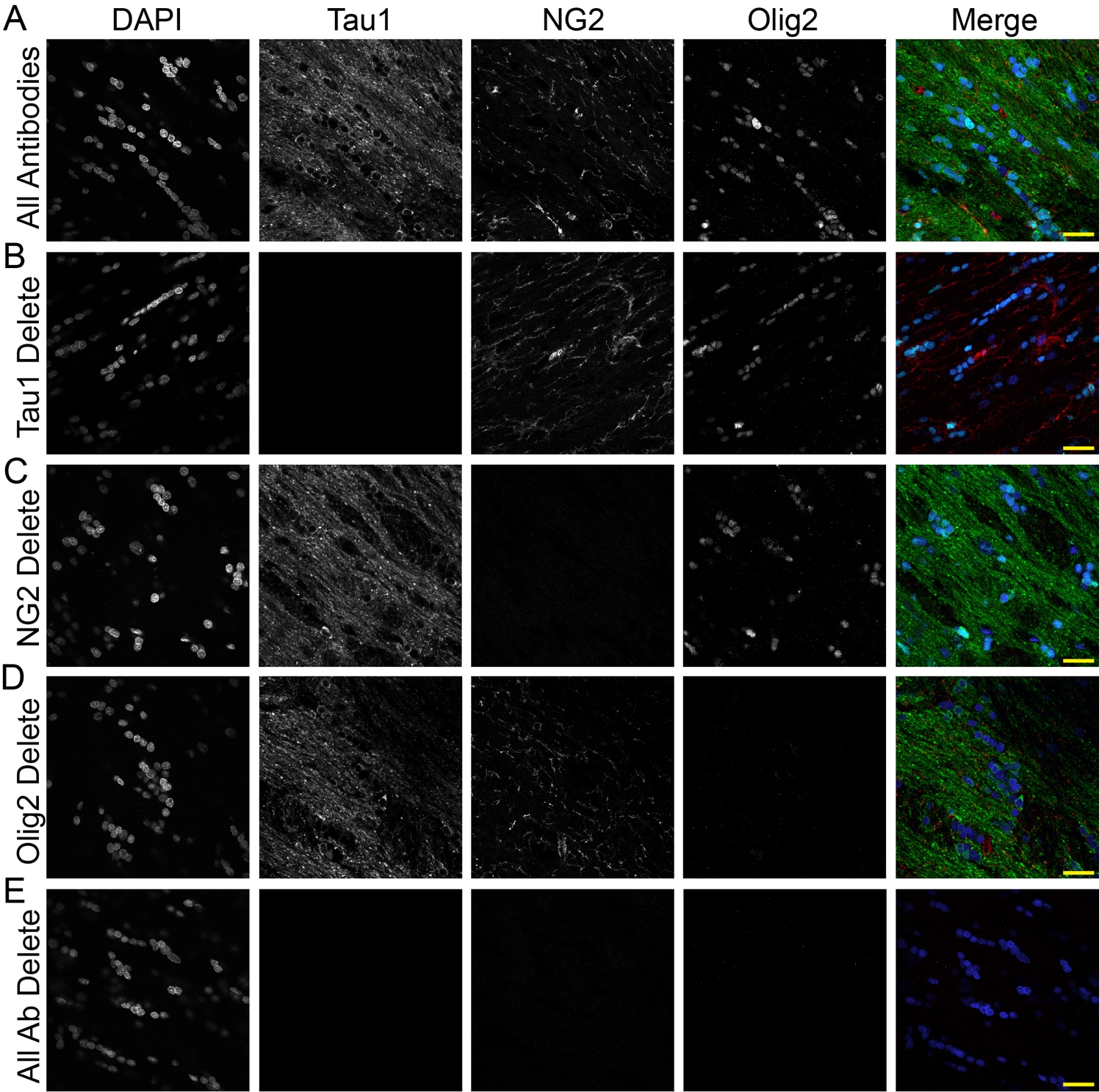
**

**Supplementary Figure 7.** Primary antibody delete controls for Tau1, NG2 and Olig2 multi-label immunofluorescence in rat tissue. **(A)** Tissue sections (corpus callosum depicted) were stained with Tau1, NG2 and Olig2 and counterstained with DAPI. **(B-E)** Sections were processed following the same procedures with the exception of omitting either Tau1 (**B**), NG2 **(C,** oligodendrocyte precursor marker**)**, Olig2 (**D**, oligodendrocyte marker) or all primary antibodies **(E)**. Note the lack of staining cross-over in individual primary deletes or non-specific signal in the full primary delete demonstrating specific detection of each antibody. Scale bars are 25 μm.

**
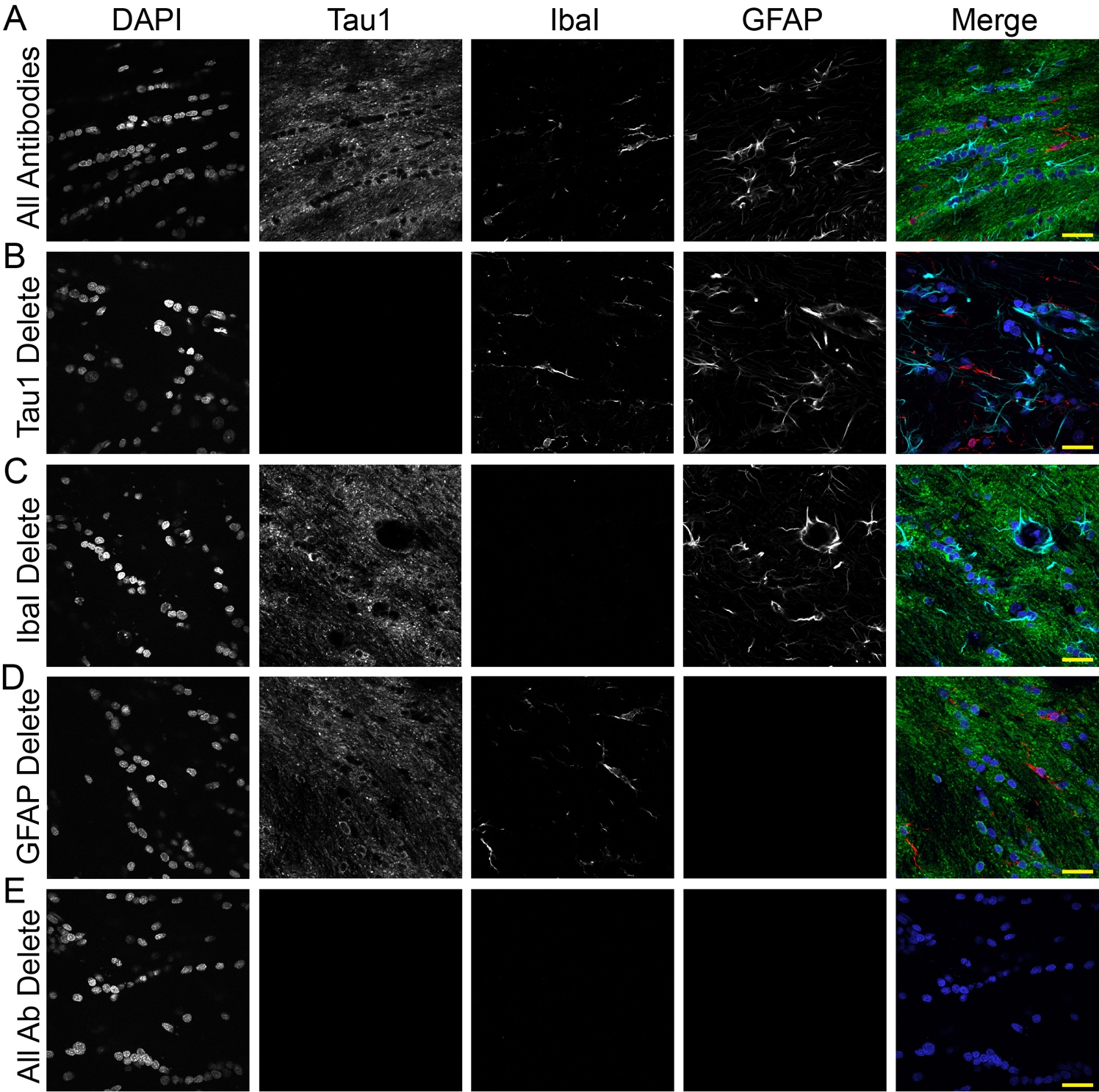
**

**Supplementary Figure 8.** Primary antibody delete controls for Tau1, IbaI and GFAP multi-label immunofluorescence in rat tissue. **(A)** Tissue sections (corpus callosum depicted) were stained with Tau1, IbaI and GFAP and counterstained with DAPI. **(B-E)** Sections were processed following the same procedures with the exception of omitting either Tau1 (**B**), IbaI **(C,** microglia marker**)**, GFAP (**D**, astrocyte marker) or all primary antibodies **(E)**. Note the lack of staining cross-over in individual primary deletes or non-specific signal in the full primary delete demonstrating specific detection of each antibody. Scale bars are 25 μm.


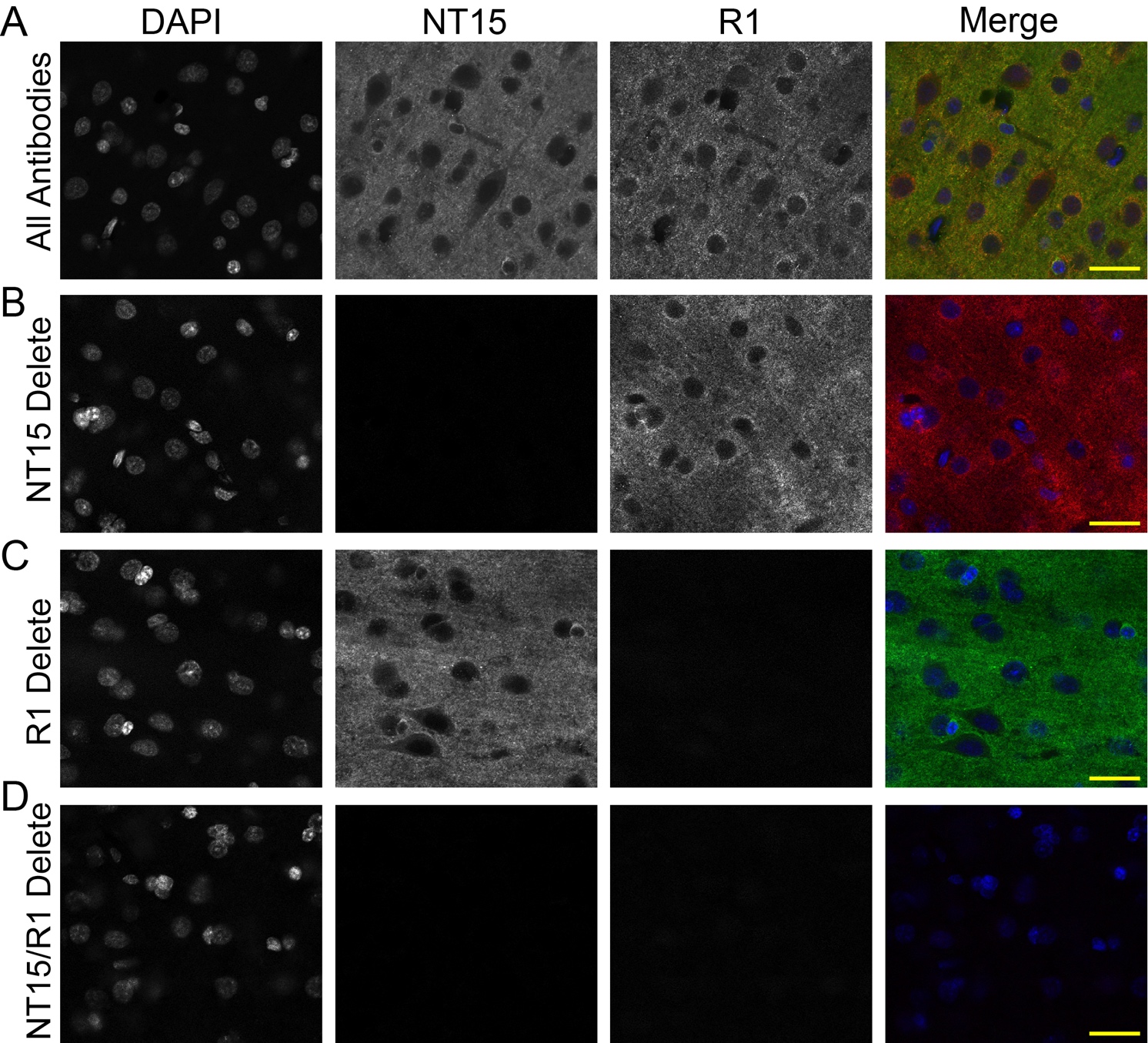


**Supplementary Figure 9.** Primary antibody delete controls for NT15 and R1 multi-label immunofluorescence in monkey tissue. **(A)** Tissue sections (cortex depicted) were stained with NT15, R1 and counterstained with DAPI. **(B-D)** Sections were processed following the same procedures with the exception of omitting either NT15 **(B)**, R1 **(C)** or both tau primary antibodies **(D)**. Note the lack of staining cross-over in individual primary deletes or non-specific signal in the full primary delete demonstrating specific detection of each tau antibody. As in the hippocampus, NT15 and R1 label somatodendritic tau in neurons. Scale bars are 50 μm.

**
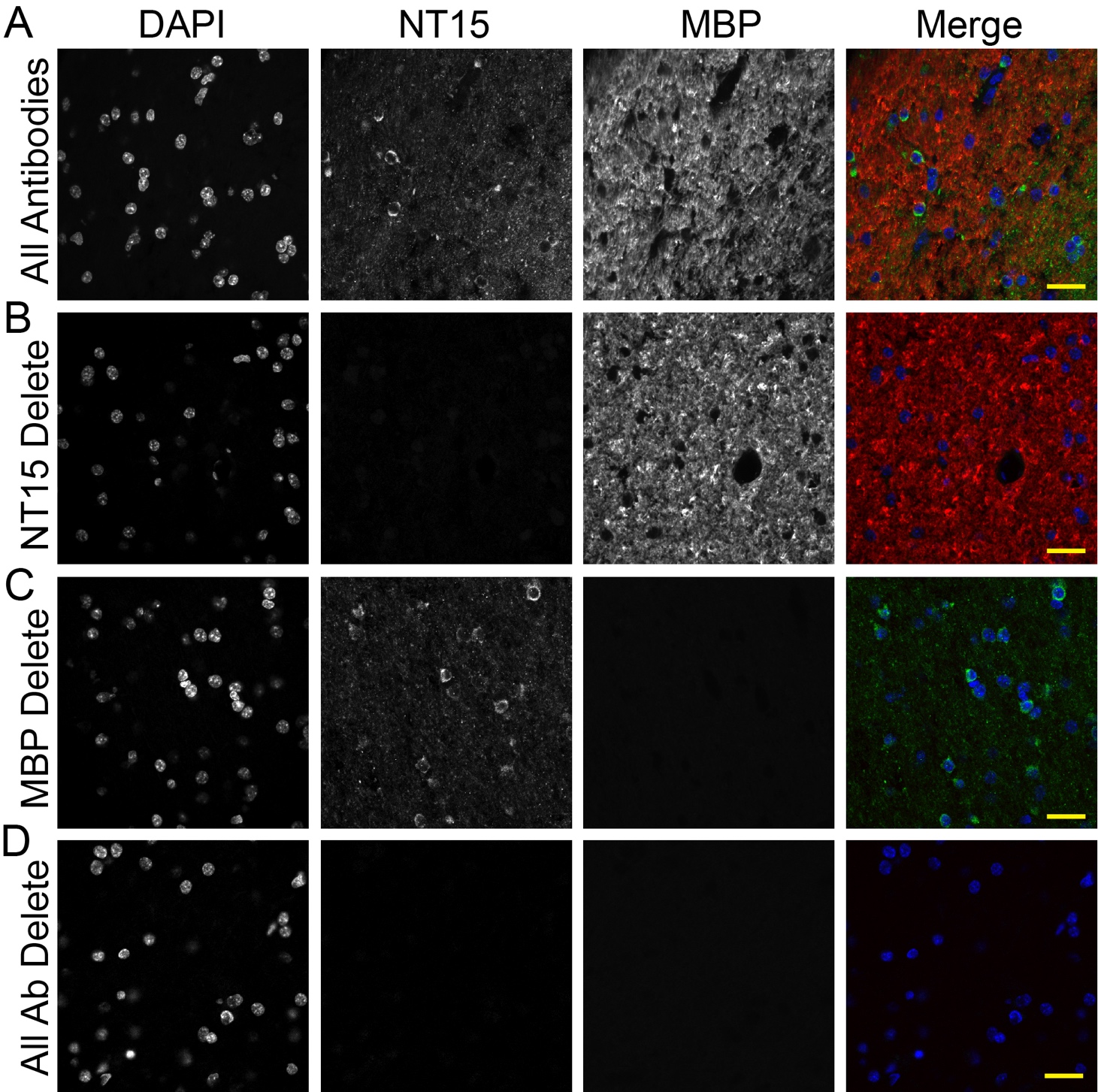
**

**Supplementary Figure 10.** Primary antibody delete controls for NT15 and MBP multi-label immunofluorescence in monkey tissue. **(A)** Tissue sections (cortex depicted) were stained with NT15, MBP and counterstained with DAPI. **(B-D)** Sections were processed following the same procedures with the exception of omitting either NT15 **(B)**, MBP **(C**, mature oligodendrocyte marker**)** or all primary antibodies **(D)**. Note the lack of staining cross-over in individual primary deletes or non-specific signal in the full primary delete demonstrating specific detection of each antibody. Scale bars are 25 μm.


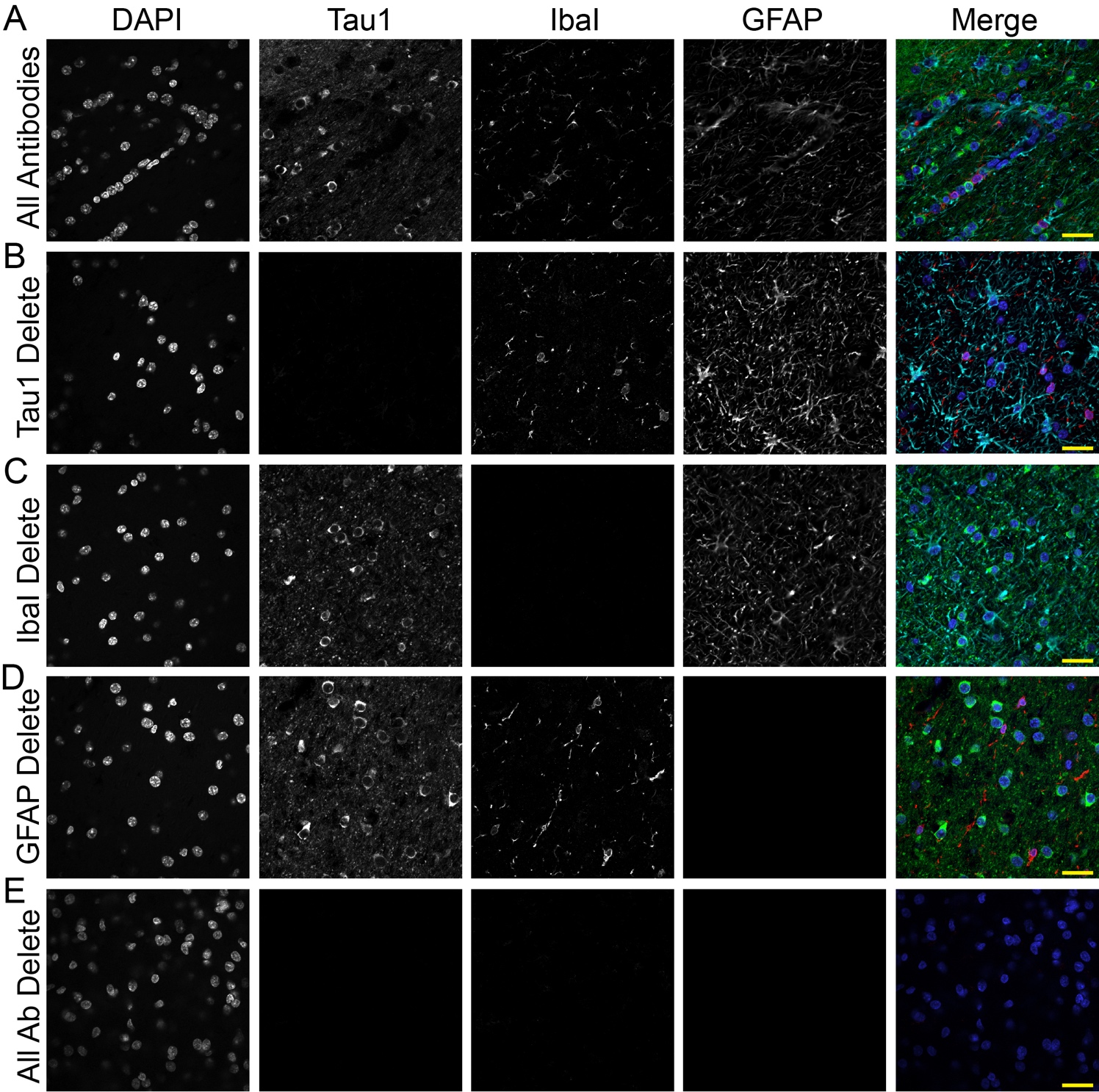


**Supplementary Figure 11.** Primary antibody delete controls for Tau1, IbaI and GFAP multi-label immunofluorescence in monkey tissue. **(A)** Tissue sections (cortex depicted) were stained with Tau1, IbaI, GFAP and counterstained with DAPI. **(B-E)** Sections were processed following the same procedures with the exception of omitting either Tau1 **(B)**, IbaI **(C**, microglia marker**)**, GFAP **(D**, astrocyte marker**)** or all primary antibodies **(E)**. Note the lack of staining cross-over in individual primary deletes or non-specific signal in the full primary delete demonstrating specific detection of each antibody. Scale bars are 25 μm.
